# Cyanogenesis in Arthropods: From Chemical Warfare to Nuptial Gifts

**DOI:** 10.3390/insects9020051

**Published:** 2018-05-03

**Authors:** Mika Zagrobelny, Érika Cristina Pinheiro de Castro, Birger Lindberg Møller, Søren Bak

**Affiliations:** 1 Plant Biochemistry Laboratory, Department of Plant and Environmental Sciences, University of Copenhagen, 1871 Frederiksberg C, Denmark; blm@plen.ku.dk (B.L.M.); bak@plen.ku.dk (S.B.); 2 Department of Ecology, Federal University of Rio Grande do Norte, Natal-RN, 59078-900, Brazil; erca@plen.ku.dk; 3 VILLUM Center for Plant Plasticity, University of Copenhagen, 1871 Frederiksberg C, Denmark

**Keywords:** cyanogenic glucosides, Zygaenidae, Papilionidae, Polydesmida, cytochrome P450, UDPG-glucosyltransferase, β-glucosidase, β-cyanoalanine synthase

## Abstract

Chemical defences are key components in insect–plant interactions, as insects continuously learn to overcome plant defence systems by, e.g., detoxification, excretion or sequestration. Cyanogenic glucosides are natural products widespread in the plant kingdom, and also known to be present in arthropods. They are stabilised by a glucoside linkage, which is hydrolysed by the action of β-glucosidase enzymes, resulting in the release of toxic hydrogen cyanide and deterrent aldehydes or ketones. Such a binary system of components that are chemically inert when spatially separated provides an immediate defence against predators that cause tissue damage. Further roles in nitrogen metabolism and inter- and intraspecific communication has also been suggested for cyanogenic glucosides. In arthropods, cyanogenic glucosides are found in millipedes, centipedes, mites, beetles and bugs, and particularly within butterflies and moths. Cyanogenic glucosides may be even more widespread since many arthropod taxa have not yet been analysed for the presence of this class of natural products. In many instances, arthropods sequester cyanogenic glucosides or their precursors from food plants, thereby avoiding the demand for de novo biosynthesis and minimising the energy spent for defence. Nevertheless, several species of butterflies, moths and millipedes have been shown to biosynthesise cyanogenic glucosides de novo, and even more species have been hypothesised to do so. As for higher plant species, the specific steps in the pathway is catalysed by three enzymes, two cytochromes P450, a glycosyl transferase, and a general P450 oxidoreductase providing electrons to the P450s. The pathway for biosynthesis of cyanogenic glucosides in arthropods has most likely been assembled by recruitment of enzymes, which could most easily be adapted to acquire the required catalytic properties for manufacturing these compounds. The scattered phylogenetic distribution of cyanogenic glucosides in arthropods indicates that the ability to biosynthesise this class of natural products has evolved independently several times. This is corroborated by the characterised enzymes from the pathway in moths and millipedes. Since the biosynthetic pathway is hypothesised to have evolved convergently in plants as well, this would suggest that there is only one universal series of unique intermediates by which amino acids are efficiently converted into CNglcs in different Kingdoms of Life. For arthropods to handle ingestion of cyanogenic glucosides, an effective detoxification system is required. In butterflies and moths, hydrogen cyanide released from hydrolysis of cyanogenic glucosides is mainly detoxified by β-cyanoalanine synthase, while other arthropods use the enzyme rhodanese. The storage of cyanogenic glucosides and spatially separated hydrolytic enzymes (β-glucosidases and α-hydroxynitrile lyases) are important for an effective hydrogen cyanide release for defensive purposes. Accordingly, such hydrolytic enzymes are also present in many cyanogenic arthropods, and spatial separation has been shown in a few species. Although much knowledge regarding presence, biosynthesis, hydrolysis and detoxification of cyanogenic glucosides in arthropods has emerged in recent years, many exciting unanswered questions remain regarding the distribution, roles apart from defence, and convergent evolution of the metabolic pathways involved.

## 1. Introduction

Plants and herbivores have co-evolved in a constant chemical warfare. An important element in their interactions is the ability to produce and handle bioactive natural products. Plants produce many low molecular mass bioactive compounds to oppose attacks from herbivorous insects, and they can vary the production of defence compounds depending on the biotic and abiotic challenges in their surroundings [1]. In turn, herbivorous insects have developed strategies to circumvent and manipulate all the plant chemical defence systems they encounter by, for example, detoxification, excretion or sequestration [2,3]. The ability of an herbivore to successfully avoid the chemical defence system of a plant species results in an advantageous niche with reduced numbers of competitors for the herbivore. Some herbivores can synthesise the same or similar defence compounds as are present in their food plant, which may have facilitated the original colonisation of the food plant. The ability to sequester a specific bioactive natural product from food plants may be less costly for an herbivore energy wise in comparison to biosynthesising it de novo [4]. The ability may later evolve to include additional interactions and alternative uses which are very beneficial for the herbivores.

Cyanogenic glucosides (CNglcs) are important natural products in the chemical warfare between plants and arthropods. More than 60 different CNglc structures are known, and the compounds are present in more than 2650 different plant species [5,6,7,8,9]. In animals, these compounds appear to be restricted to arthropods [10] and are only common within polydesmid millipedes [11] and Lepidoptera [12]. CNglcs are β-glucosides of α-hydroxynitriles derived from the aliphatic protein amino acids l-valine, l-isoleucine and l-leucine; from the aromatic amino acids l-phenylalanine and l-tyrosine; or from the cyclopentenoid non-protein amino acid 2-(2′-cyclopentenyl)-glycine. Consequently, CNglcs are defined as aliphatic, aromatic or cyclopentenoid, based on their parent amino acids. The aromatic CNglc prunasin is synthesised from phenylalanine [13], while cyclopentenoid CNglcs are derived from cyclopentenyl glycine [14]. The two aliphatic CNglcs, linamarin and lotaustralin, are derived from valine and isoleucine, respectively, and usually co-occur due to the ability of the biosynthetic enzymes to use both structurally related amino acids and intermediates formed as substrates. Linamarin and lotaustralin are the most abundant CNglcs in plants and arthropods [15].

CNglcs act as feeding deterrents on herbivores and predators perhaps in part due to their bitter taste [16]. However, CNglcs also release hydrogen cyanide (HCN) when they are enzymatically hydrolysed, and this is a toxic substance, mainly due to its inhibitory effect on the terminal cytochrome oxidase in the mitochondrial respiratory pathway [17]. Apart from their role in defence, CNglcs serve as endogenous repositories of reduced carbon and nitrogen in plants [18,19,20]. Two endogenous pathways for recycling of auto-toxic CNglcs have been demonstrated in sorghum, almonds and cassava and the first enzyme involved has been identified as a glutathione *S*-transferase [18,21]. Endogenous pathways to retrieve ammonia fromCNglcs without release of toxic hydrogen cyanide have been hypothesised to operate in some insects [4]. Furthermore, mandelonitrile, a cyanogenic compound from peach, has been shown to be metabolised into the plant hormone salicylic acid, important for diverse biological processes [22]. Thus, natural products are intimately linked to primary metabolism in plants, and similar mechanisms could be envisioned for arthropods. In plants, CNglcs have been reported to be stored apart from the enzymes capable of hydrolysing them [23,24,25]. When a plant tissue containing CNglcs is disrupted by, for example, herbivore attack, the CNglcs are brought into contact with hydrolysing enzymes causing release of HCN, an aldehyde or ketone, and glucose [26,27]. In arthropods, CNglcs are also stored apart from the hydrolysing enzymes. As in plants, the two-component defence system is activated upon attack [11,28]. This binary system provides plants and arthropods with both a deterrent effect on casual predators perhaps due to the bitter taste of CNglcs, and an immediate chemical defence response based on HCN toward predators and pathogens that cause tissue damage.

In this review, we first summarise the biochemical mechanisms of CNglc biosynthesis, hydrolyses and recycling, and discuss the enzymes involved in these processes. We then systematically review the presence of HCN and CNglcs in all Arthropod taxa where these compounds have been found. In the last part of the review, we outline roles of CNglcs beyond chemical defence, e.g. as pheromones.

## 2. Biosynthesis of Cyanogenic Glucosides

**Figure 1:**
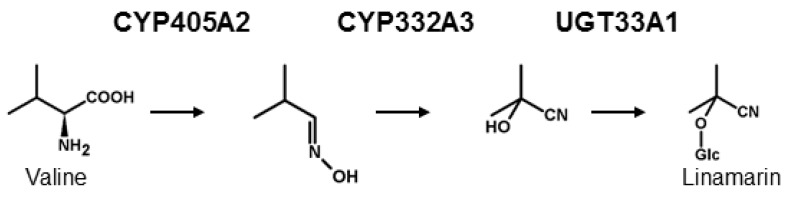
Biosynthesis of CNglcs exemplified by the aliphatic CNglc linamarin and the characterised enzymes from *Z. filipendulae*.

From a biochemical point of view, the de novo biosynthesis of CNglcs has been shown to follow a general pattern in many plant species and in the moth *Zygaena filipendulae*, although the genes encoding the enzymes in the pathway have evolved convergently in these two groups [5,29,30] (Figure 1). The first committed enzyme in CNglc biosynthesis is a cytochrome P450 [29,31], catalysing two sequential *N*-hydroxylations of the parent amino acid followed by a dehydration, and a decarboxylation reaction to produce the corresponding *E*-aldoxime. The *E*-aldoxime is subsequently converted into the *Z*-aldoxime [32], which via a dehydration reaction and C-hydroxylation gives rise to the formation of an α-hydroxynitrile (cyanohydrin). These reactions are catalysed by a second cytochrome P450 [29,33]. Finally, the cyanohydrin moiety is glycosylated by a UDPG-dependent glycosyltransferase (UGT) [29,34]. The details concerning the *E*- and *Z*- aldoxime have only been shown in plants, but the mechanism is expected to be similar in insects. In *Z. filipendulae*, de novo biosynthesis of CNglcs is catalysed by the enzymes CYP405A2, CYP332A3 and UGT33A1 [29]. All heliconiine butterflies are thought to biosynthesise CNglcs de novo as well, and at least *Heliconius melpomene* share the same parent amino acids and intermediates as found in the pathway in *Z. filipendulae* [35]. Since genes homologous to *CYP405A2* and *CYP332A3* have been found in the genome of *H. melpomene* [36] (*CYP405A4*, *CYP405A5*, *CYP405A6* and *CYP332A1*) and are also present in several other *Heliconius* species as well as quite widespread within Lepidoptera [37], the biosynthesis of CNglcs could be orthologous within this order. In the millipede *Chamberlinius hualienensis*, CYP3201B1 was shown to catalyse the conversion of phenylacetonitrile into mandelonitrile, the cyanogenic component of its defensive secretion [38]. This enzyme is not orthologous to CYP332A3 and therefore represents a third convergent evolution of the biosynthesis of cyanogenic components.

The first committed step in a biosynthetic pathway is typically catalysed by an enzyme with high substrate specificity [39] to narrow down the number of available substrates for the later enzymes in the same pathway. These later enzymes may therefore have a wider substrate specificity providing overall metabolic flexibility, but nonetheless desired specificity when they are associated with other biosynthetic enzymes [40]. This has been found for enzymes involved in the biosynthesis of CNglcs in both plants [41] and insects [42]. In plants containing the CNglcs linamarin and lotaustralin, the relative amounts of the two CNglcs is predominantly determined by the preference of the first enzyme in the pathway for either valine or isoleucine (the parent amino acids) or by the in vivo availability of these as substrates. This is illustrated by the stoichiometric amounts of linamarin and lotaustralin formed in vitro using CYP79D1 and CYP79D2 from cassava (*Manihot esculenta*) [43], whereas the ratio between linamarin and lotaustralin is 93 to 7 when studied in vivo [44,45]. In the legume *Lotus japonicus*, the in vivo ratio between linamarin and lotaustralin is 1:10, probably due to the catalytic efficiency of the first enzyme in the pathway being six times greater for isoleucine than for valine [46]. In *Zygaena* species, administration of radiolabelled valine and isoleucine, demonstrated preferential incorporation into linamarin compared to lotaustralin [35]. However, CYP405A2 from *Z. filipendulae* had a higher preference for isoleucine than valine in vitro [29], which results in higher production of lotaustralin. The ratio of linamarin:lotaustralin also changes during the *Zygaena* sp. life cycle, possibly due to the amount of sequestration and turn-over taking place, as well as the preference of the first enzyme in the pathway [47]. In heliconiine butterflies, including *H. melpomene*, the linamarin:lotaustralin ratio is higher than in *Zygaena* moths. In both *Zygaena* and *Heliconius*, the linamarin:lotaustralin ratio and overall content varies throughout their life-cycle, and is to some extent influenced by their larval food plant [35,42,48,49]. Accordingly, sequestration of CNglcs has an effect on the activity of the biosynthetic pathway.

The CNglc biosynthetic pathway may be hypothesised as having evolved by initial recruitment and selection of the first enzyme in the pathway. As more sequencing data have become available, it has become apparent that *CYP79*s are present in all sequenced or otherwise analysed eudicots and non-eudicot angiosperm plants [50]. All flowering plants thus have the CYP79 blueprint to, theoretically, produce oximes [51]. The two subsequent enzymes in the CNglc biosynthetic pathway have been shown to be more promiscuous, probably retaining some other functions apart from the CNglc biosynthetic pathway [42,52]. *CYP332* genes have a wider distribution within Lepidoptera than *CYP405s* and have been proposed to be involved in the detoxification of ingested plant compounds [37]. The anabolism of aldoximes probably evolved first in species not producing CNglcs, as part of the biosynthesis of the phytohormone auxin from tryptophan [37], a pathway also found in many insects [53,54]. This insect pathway involves indole acetaldoxime (IAOx) as intermediate, which have been shown to be an intermediate for auxin biosynthesis in *Arabidopsis* sp. [55,56]. The last enzyme in the pathway, the UGT, may not be orthologous between butterflies and moths [37], signifying that perhaps several different UGTs were able to perform this function in the common ancestor of butterflies and moths.

**Figure 2:**
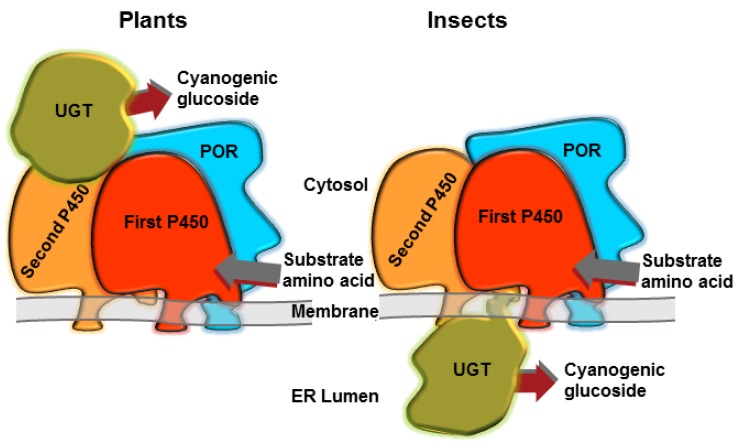
Hypothetical representations of metabolons for the biosynthesis of CNglcs in plants and insects. Adapted from [29]. The components are not necessarily present in stoichiometric amounts in vivo.

The intermediates of CNglc biosynthesis (oximes and cyanohydrins) are unstable volatiles, which could be partly lost during production and could even elicit toxic effects within the organism. To avoid this, the biosynthesis of the CNglc dhurrin is highly channelled in the plant *Sorghum bicolor* [57], with the three enzymes involved in the pathway forming a dynamic metabolon (Figure 2) [58,59]. Metabolon formation reduces the risk of undesired metabolic cross-talk and improves catalytic efficiency by bringing co-operating active sites into close proximity, facilitating swift delivery of intermediates from one active site to the next, and avoiding escape of toxic intermediates [59,60]. Dynamic metabolons additionally provide possibilities for swift redirection of metabolism by exchange of the incorporated enzyme components resulting in an altered product output as might be demanded by environmental challenges. It is not known if the CNglc biosynthesis in arthropods also form a metabolon (Figure 2), but metabolons have been demonstrated to be involved in, e.g., melanin synthesis in insects [61], and interactions between P450s and UGTs have been demonstrated in mammals [62,63]. Radiolabelled valine and isoleucine primarily ended up in linamarin and lotaustralin in *Z. filipendulae* larvae, while no toxic intermediates in the CNglc biosynthetic pathway were found [64]. Furthermore, the genes involved in the pathway are expressed and localised to the same tissues in *Z. filipendulae* [42], so it is likely that CNglc biosynthesis is carried out in a metabolon at least in moths.

## 3. Hydrolysis of Cyanogenic Glucosides

**Figure 3:**
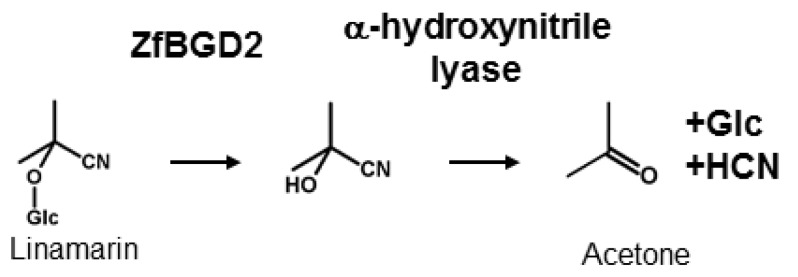
Hydrolysis of CNglcs exemplified by the aliphatic CNglc linamarin and the characterised β-glucosidase and envisioned α-hydroxynitrile lyase enzymes from *Z. filipendulae*.

CNglcs are not toxic when intact but have to be activated to release HCN for defence, as well as to recycle nitrogen and glucose into other metabolic processes. This activation is catalysed by a β-glucosidase that removes the glucose residue stabilising the CNglc structure, converting it into the corresponding α-hydroxynitrile, which spontaneously dissociates into a sugar, a keto compound, and HCN at pH values above 6 (Figure 3). The dissociation reaction is catalysed by an α-hydroxynitrile lyase at lower pH values. β-glucosidases have been characterised from many cyanogenic plant species [65] and are generally very stable with acidic pH optima (pH 5–6) [66,67]. α-Hydroxynitrile lyases have only been characterised from a few plant species [68], but they appear to co-localise with the CNglc-hydrolysing β-glucosidases [69]. The β-glucosidase responsible for hydrolysing linamarin and lotaustralin in *Z. filipendulae* was recently characterised (ZfBGD2) and this gene is expressed in the haemocyte-part of the haemolymph (free floating cells), while the enzyme activity is restricted to the haemoplasma in which the CNglcs are also present [28]. The enzyme probably functions as a dimer, has characteristics similar to plant β-glucosidases, and exhibits higher activity against lotaustralin than linamarin [70]. It is still not known how the enzyme activity is regulated to avoid intoxication of the insect, but there are indications that it has to be bound to another protein to gain full activity. One such candidate could be the α-hydroxynitrile lyase needed for full rapid release of HCN, since this enzyme activity appear tightly bound to the β-glucosidase when attempting to purify it from *Z. filipendulae* haemolymph (Zagrobelny, unpublished). No α-hydroxynitrile lyases have been adequately characterised from insects yet, but a partly purified α-hydroxynitrile lyase was obtained from the haemolymph of *Zygaena trifolii* [71]. The enzyme is supposedly a dimer probably containing a flavin group as co-factor.

Another α-hydroxynitrile lyase (ChuaMOX) was reported from the cyanogenic millipede *Chamberlinius hualienensis*. This enzyme catalyses dissociation of its substrate mandelonitrile to release HCN for defence [72]. It binds a flavin group and was shown to be glycosylated [72]. The enzyme seems to be spatially separated from (R)-mandelonitrile, like the CNglc hydrolysing enzymes from *Z. filipendulae* and plants, which highlights the similarities in cyanide-based defence in these different kingdoms of life. Although the hydrolytic pathways of CNglcs in plants and arthropods are highly similar, comprising enzymes from the same families, they have clearly evolved convergently, similar to the biosynthetic pathways.

## 4. Detoxification of HCN Released from Cyanogenic Glucosides

**Figure 4:**
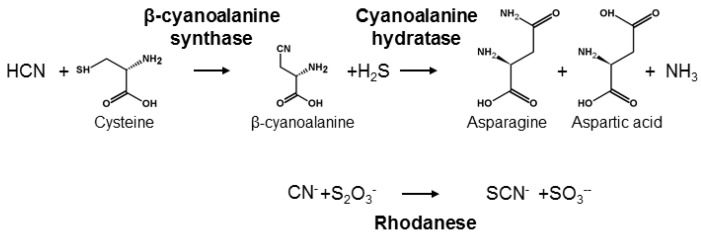
Detoxification of CNglcs.

HCN is poisonous for most living organisms because it acts as a strong inhibitor of the respiratory chain and also impairs several other metabolic pathways [73]. Thus, living organisms have evolved different mechanisms to detoxify HCN and avoid its deleterious effects [7]. One mechanism is catalysed by β-cyanoalanine synthase and requires the formation of the amino acid β-cyanoalanine from cysteine or serine (Figure 4). In plants, β-cyanoalanine is further converted into asparagine or aspartate and ammonia by cyanoalanine hydratases [74]. β-cyanoalanine synthase activity plays a central role in detoxification of HCN formed in stoichiometric amounts with the plant hormone ethylene [75] and is therefore universal in plants [76]. Accordingly, herbivores feeding on plant tissue are always exposed to minute amounts of HCN. β-cyanoalanine accumulation in some plants may furthermore serve to deter predators since it is a potent neurotoxin [77]. In the animal kingdom, only nematodes and arthropods contain β-cyanoalanine synthase. In arthropods the enzyme is present in a broad range of Lepidopteran species. This includes species containing CNglcs, such as Zygaenidae, but also acyanogenic species [78,79]. In the butterfly *H. melpomone* (Papilionoidea), β-cyanoalanine synthase activity is only present in feeding larval stages, corresponding to the developmental stages at which detoxification of ingested cyanogenic plant material is in demand [78,80]. The β-cyanoalanine synthase enzyme in the spider mite *Tetranychus urticae* was characterised [81] and shown to result from a horizontal transfer from a bacterium. Homologous genes are present in the genomes of many lepidopterans [82], and especially in several butterflies where this gene is triplicated [83]. It is not known if the presence of this enzyme in this clade resulted from one or more horizontal gene transfers from bacteria in close association with ancestral arthropods [84].

Another mechanism for HCN detoxification can be found in many bacteria, plants and animals, including vertebrates, and is catalysed by rhodaneses [85] (Figure 4). Rhodaneses transfer a sulphur from a thiosulphate to a HCN using cysteine as sulphur carrier, producing thiocyanide and sulphite. Accordingly, rhodanese is also called thiosulphate:cyanide sulphurtransferase. In addition to HCN detoxification, rhodaneses serve a variety of functions, the most important of which is to donate sulphur to proteins [86,87]. Rhodanese activity is constitutive throughout the life-cycle of several insects, and in *Spodoptera eridania*, it is not enhanced by the presence of HCN or other breakdown products of CNglcs [88]. Furthermore, some insect species that feed on cyanogenic plants seems to lack this enzyme while many species eating a non-cyanogenic diet have it [86,87]. Consequently, rhodaneses are probably not directly linked to HCN detoxification in insects, but play a major role in primary metabolism rather than in detoxification of allelochemicals [86,87].

The enzymes involved in biosynthesis, recycling and hydrolysis of CNglcs all belong to multigene families which are found in most or all living organisms. The enzymes are characterised by being involved in important functions as housekeeping enzymes in primary metabolism as well as in detoxification of xenobiotics and allelochemicals and in the synthesis and turn-over of bioactive natural compounds. In many cases, new enzyme functions appear due to a few or sometimes a single amino acid substitution, as observed for plant CYP79s [5] and β-glucosidases in maize and sorghum [89]. Enzymes from these families are therefore well suited for recruitment into new functions such as the evolution of CNglc metabolism.

## 5. Cyanogenic Glucosides in Food Plants

Cyanogenesis is one of the most widely distributed chemical defences in the plant kingdom, since >2600 species produce CNglcs for defence against herbivores [8]. Since CNglcs are nitrogen-based, this type of defence is considered costly for plants [90]. Consequently, natural populations of cyanogenic plants vary a lot in their content of CNglcs [47,91,92,93], and variation is especially striking with respect to leaf age and environmental conditions [16,94]. Biosynthesis of CNglcs predominantly takes place in young and developing tissues [46,94], and consequently the CNglc levels found in older plant parts often decrease because biosynthesis proceeds at a lower rate than turnover or cannot keep up with the net gain in total biomass [95]. Consequently, a plant which is safe for an herbivore under one set of conditions, may be toxic or lethal under other conditions [94].

**Figure 5:**
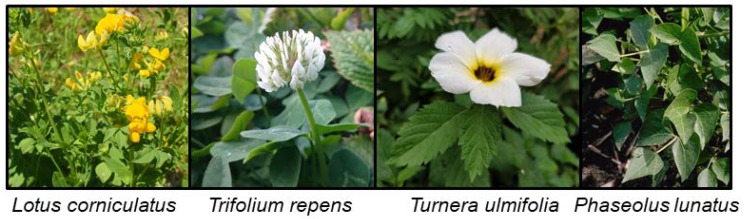
*L. corniculatus* photo by Mika Zagrobelny, *T. repens* and *T. ulmifolia* photos by Érika Cristina Pinheiro de Castro, and *P. lunatus* photo by Howard F. Schwartz (https://www.forestryimages.org/browse/detail.cfm?imgnum=5357638).

The presence of the CNglcs linamarin and lotaustralin as well as cyanogenesis have been found to be polymorphic traits in several plant species (Figure 5). Thus, cyanogenic and acyanogenic individuals can be found within or between populations [96,97,98,99]. In white clover (*Trifolium repens*) these polymorphic trait differences have been shown to arise due to the presence or absence of functional enzymes involved in the synthesis and hydrolysis of CNglcs [100]. Cyanogenic genotypes of *T. repens* have lower freezing tolerance compared to acyanogenic genotypes, possibly due to frost induced autotoxicity when plant cell disruption results in exposure of CNglcs to hydrolysing enzymes [101]. The frequency of cyanogenic plants among *T. repens* populations steadily decrease at higher elevations [102], which could be due both to decreasing temperature, but also to lower herbivore pressure in colder climates. The opposite was observed for bird’s-foot trefoil (*Lotus corniculatus*) where plants at higher elevations contained increased levels of CNglcs, possibly to gain better protection against herbivores in a low resource habitat [103]. Cyanogenic *T. repens* grows faster and gains a greater flower head mass during water stress compared to acyanogenic plants [104], perhaps due to the role of CNglcs as nitrogen transport and storage molecules [18,19,20]. Increased soil salinity also leads to an upregulation of CNglcs in *T. repens* [105]. Natural products from plants have generally been hypothesised to prevent damage caused by radicals produced as a result of different abiotic stresses [106], so cyanogenic plants may have an advantage compared to acyanogenic ones during such conditions.

Many generalist herbivores tolerate low levels of CNglcs in their diet but are intoxicated if forced to feed on plants with a high CNglc content. Consequently, generalist herbivores typically avoid feeding on highly cyanogenic plants, while specialist herbivores are specifically attracted to such plants. Accordingly, acyanogenic *L. corniculatus* plants are grazed more heavily by generalist herbivores than cyanogenic ones, but this negative impact is partly compensated by more vigorous growth of acyanogenic plants [107,108]. The same effect was observed in lima bean (*Phaseolus lunatus)* and *T. repens* where plants with low amounts of CNglcs produced more biomass and more seeds [97,109]. Highly cyanogenic *P. lunatus* plants, on the other hand, were a lot less affected by herbivory than plants with few CNglcs [97]. Cyanogenic plants would profit from the presence of CNglcs in years with many generalist insects and few specialist insects, and perhaps by having increased fitness under abiotic stresses. Acyanogenic plants would benefit in the absence of generalist herbivores by saving resource allocation to CNglc synthesis [108]. Consequently, selection would probably maintain both genotypes in plants to ensure the plasticity needed for diversified and optimised responses to biotic and abiotic challenges.

Contrary to the widespread aliphatic CNglcs, cyclopentenyl CNglcs are only produced by a few closely related plant families, where Passifloraceae and Turneraceae are the most common. Polymorphism in cyanogenesis has been observed in *Turnera ulmifolia*, where “acyanogenic” populations only produce CNglcs as seedlings [110]. This developmental selection is probably associated with their major herbivore, the butterfly *Euptoieta hegesia*, which sequester cyclopentenyl CNglcs from these plants [110]. Although the absence of cyanogenesis do not interfere with the oviposition preferences of *E. hegesia*, they are more vulnerable to lizard attacks when reared on acyanogenic plants. Additionally, cyanogenesis in *T. ulmifolia* is negatively correlated with dry seasons and flower numbers, which could indicate that the plants stop manufacturing CNglcs when resources are scarce [110,111], confirming that CNglc biosynthesis is costly for the plant.

The crucifer specialist flea beetles, *Phyllotreta nemorum*, have previously been shown to ingest less and have higher mortality when feeding on cyanogenic plants [112]. However, several generalist Lepidopterans forced to feed on cyanogenic diets did not display any adverse effects from feeding on these plants [113]. In those cases, a major proportion of the ingested CNglcs were recovered in the frass of the insects, indicating that they have developed special adaptation mechanisms to keep CNglcs intact until excretion [113]. Consequently, deterrent effects of CNglcs observed in the field might in some cases be ascribed to their bitter taste [16] rather than HCN emission, at least when observed in Lepidopteran species.

## 6. Cyanogenic Glucosides in Arthropods

**Figure 6:**
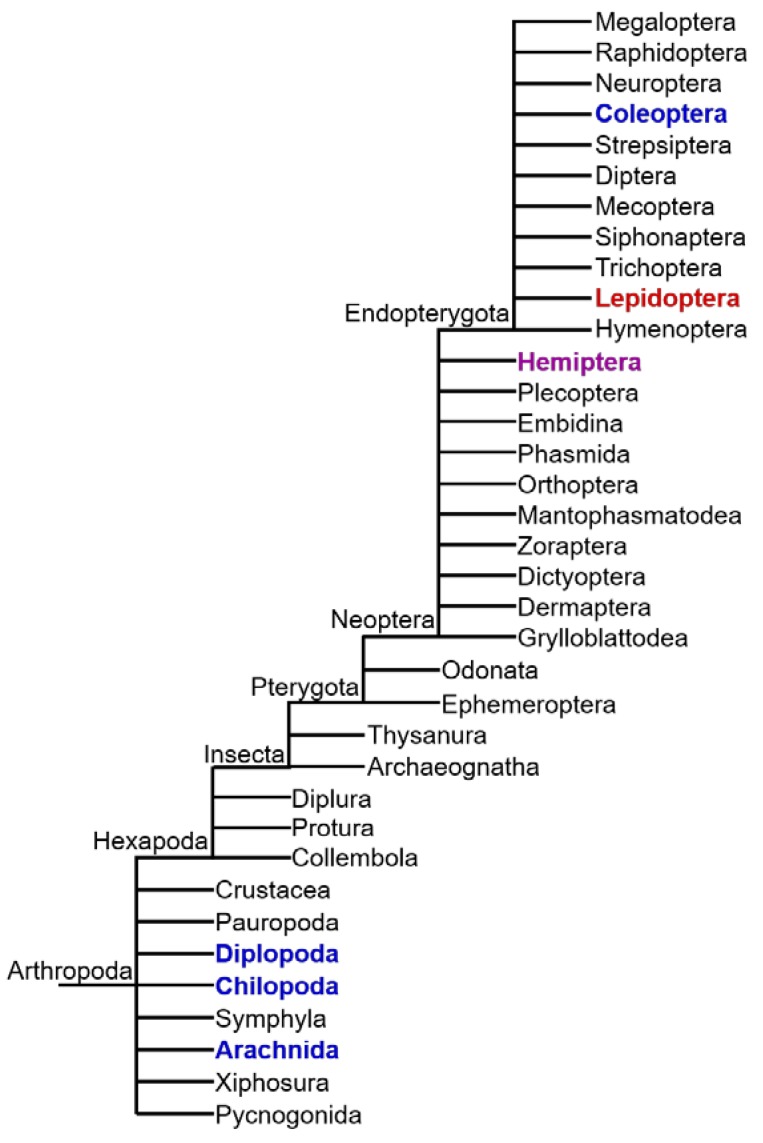
Schematic representation of Arthropod phylogeny with clades containing species carrying aliphatic (**red**) and aromatic (**blue**) CNglcs, as well as other cyanogenic compounds (**purple**). Cyclopentenoid CNglcs are only found within Heliconiinae butterflies (Lepidoptera). Based on Tree of Life web project (http://tolweb.org). Only extant clades are included.

**Figure 7:**
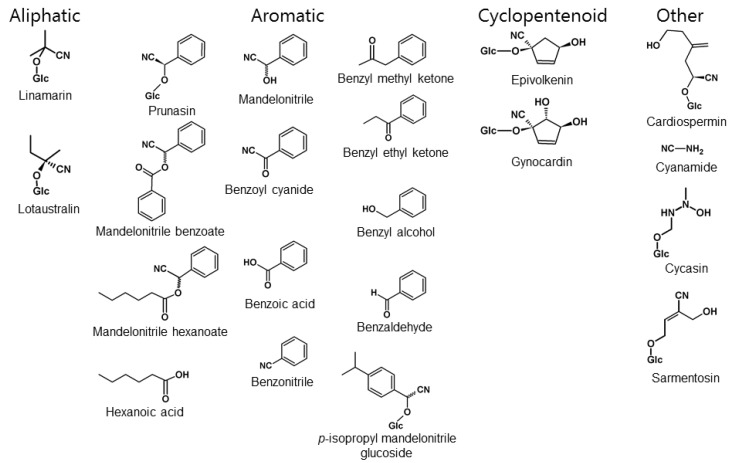
Cyanogenic compounds or derivatives thereof discussed in this paper sorted into the aliphatic, aromatic or cyclopentenoid groups of CNglcs or a group of other cyanogenic compounds.

Although many animal species produce or sequester toxic natural compounds, the presence of CNglcs appears to be restricted to Arthropoda [10]. CNglcs are found in species within Chilopoda (centipedes), Diplopoda (millipedes), Arachnida (mites) and Insecta [80] (Figure 6). In Insecta, CNglcs have thus far been found only in the orders Hemiptera (bugs, aphids, etc.), Coleoptera (beetles), and Lepidoptera (butterflies and moths) [12]. CNglcs may be more widespread within Arthropoda since many taxa have not been properly tested. Chilopoda, Diplopoda, Arachnida and Coleoptera contain only aromatic CNglcs, while Lepidoptera contain mainly aliphatic CNglcs, and in a few cases aromatic and cyclopentenoid CNglcs as well (Figure 7). Since cyanogenesis shows a very scattered phylogenetic distribution within arthropods, it has probably evolved several times in this phylum. Species of arthropods containing CNglcs are thought to use the compounds mainly for defence, like plants, although other roles have been suggested, especially within butterflies and moths (discussed in Section 9).

Contrary to plants, much of the data about cyanogenic arthropods available in the literature were obtained before 1980 using much less precise and sensitive methods compared to those available today. Extraction methods included experimental conditions where most constituents involved in cyanogenesis would decompose, and consequently the chemical structure of the constituent giving rise to HCN formation could often not be determined. However, new evidence from millipedes, butterflies and moths has cast new light on the presence, function and evolution of CNglcs in arthropods. With the experimental limitations concerning older data in mind, an overview of the present knowledge on cyanogenesis in arthropods is presented below (summarised in Table 1) with special emphasis on butterflies and moths.

### 6.1. Chilopoda

**Figure 8:**
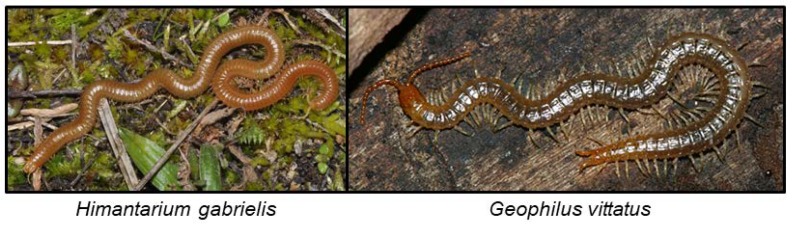
*H. gabrielis* photo by Pascal Dubois (https://www.galerie-insecte.org/galerie/esp-page.php?genre=Himantarium&espece=gabrielis) and *G. vittatus* photo by Tom Murray (https://bugguide.net/node/view/16621).

The majority of centipedes examined produce a proteinaceous defensive secretion stored in segmental glands, which is discharged when the centipede is disturbed or challenged. Some centipedes of the order Geophilomorpha (in the families Linotaeniidae, Geophilidae and Himantariidae) contain cyanogenic constituents in their secretions [116]. The defensive secretions from *Asanada* sp. contain HCN [114], and *Pachymerium ferrugineum* was shown to be cyanogenic, but the cyanogenic constituents were not identified. The secretion of *Himantarium gabrielis* (Figure 8) contained HCN, benzaldehyde, benzoyl cyanide, benzyl cyanide, mandelonitrile and mandelonitrile benzoate among other things [116], and *Geophilus vittatus* (Figure 8) similarly contained benzaldehyde, benzoic acid, benzoyl cyanide, HCN and mandelonitrile at a pH of 6–6.5 [115]. Other geophilomorphs were hypothesised to produce similar substances, and the substances were also used by females to guard their eggs. It was proposed that centipedes store mandelonitrile and benzoyl cyanide as HCN precursors [115]. Experiments showed that the sticky cyanogenic secretions made predatory ants release the centipedes, but ants still got stuck to each other or to the ground as the secretion deposited on them gradually hardened, killing them in the end [115]. In cases where the reservoir of defensive secretion was depleted, the centipedes were readily killed by the ants. The effects reported for the cyanogenic centipede secretion are similar to the defensive properties of the secretion of the Lepidopteran *Z. filipendulae* (see Section 6.6.1).

### 6.2. Diplopoda

**Figure 9:**
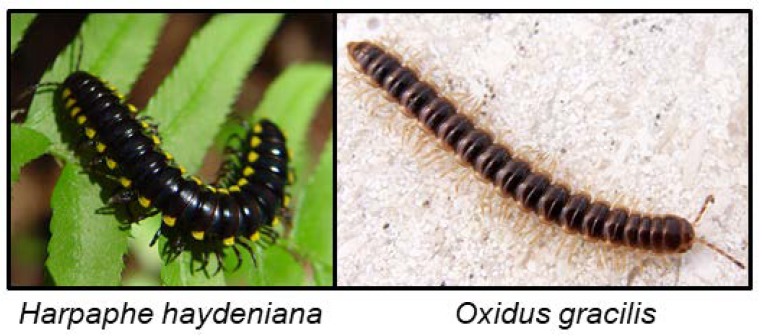
*H. haydeniana* photo by Franco Folini (https://commons.wikimedia.org/wiki/File:Harpaphe_haydeniana_002.jpg) and *O. gracilis* photo by João Coelho (https://commons.wikimedia.org/wiki/File:Oxidusgracilis.png).

Millipedes contain many different classes of defence compounds, which are all endogenously produced and not sequestered from their food sources [11]. More than 140 species have been analysed and most millipedes from the order Polydesmida so far examined are cyanogenic [11]. In most cases, the HCN arises from hydrolysis of mandelonitrile [141,152,153]. To counteract autotoxicity, millipedes have evolved a cytochrome oxidase enzyme which is highly tolerant to HCN [154]. Millipedes control the ejection of their cyanogenic secretions from glands through ozopores on their dorsal surface by increasing the haemolymph pressure or by contracting muscles nearby the glands. *Harpaphe haydeniana* (Figure 9) stores mandelonitrile in oily droplets in one section of the defence gland. The mandelonitrile is squeezed out through a reaction chamber, where it is mixed with α-hydroxynitrile lyase to release benzaldehyde and HCN [10,132]. The pH in the reaction chamber is 4, which is optimal for the activity of α-hydroxynitrile lyase, and for preventing the mandelonitrile from spontaneous disassociation as long as it is kept apart from the enzyme. The cyanogenic glands of *H. haydeniana* were also shown to contain β-glucosidase activity, and *H. haydeniana* furthermore contains β-cyanoalanine synthase and rhodanese for detoxification of the CNglc hydrolysis products [155]. When the glands containing the defensive secretions of millipedes are depleted, it may take up to four months to fully replenish the content [11], indicating that the biosynthetic pathway in millipedes is not very effective or that mandelonitrile formation is hampered by lack of the amino acid substrate phenylalanine. The cyanogenic defence components from millipedes have been shown to deter several different predators [120], but it was ineffective towards the predatory tarantula *Megaphobema mesomelas*, possibly due to the fast attack from this predator preventing release of HCN from millipedes [156]. The predator did not appear affected by the intact cyanogenic components from the ingested prey, although how it coped with the toxins was not examined in this study [156]. One supposedly acyanogenic millipede (*Niponia nodulosa*) was shown to contain cyanogenic compounds in the younger instars [117], indicating that cyanogenesis may be more widespread than hitherto expected, but also supporting the hypothesis that production of cyanogenic compounds is costly and, in some instances, reserved for the most vulnerable life stages.

The biochemical pathways for CNglc biosynthesis in *H. haydeniana* and *Oxidus gracilis* (Figure 9) involve similar or identical intermediates to those known from higher plants and insects (*N*-hydroxy amino acids, oximes, and nitriles) [10]. *O. gracilis* emit HCN and benzaldehyde [121], which probably represents mandelonitrile or prunasin as the final stored product, both produced from phenylalanine [11]. *O. gracilis* furthermore has rhodanese, which may facilitate detoxification of endogenous levels of HCN. The swarm-forming millipede *Chamberlinius hualienensis* secretes mandelonitrile, benzaldehyde and HCN in a defensive secretion due to strong muscle contractions presumably resulting in the mixing of substrate and hydrolysing enzymes [72]. *Apheloria corrugate* contains mandelonitrile stored separately from the hydrolysing enzymes. Stimulation of the millipede results in production of benzaldehyde and HCN probably due to mixing of the two components [155]. Some polydesmid millipedes produce mandelonitrile benzoate from benzoyl cyanide and benzaldehyde via a Schotten–Baumann reaction under basic conditions due to bleeding of bodily fluids which results in rapid HCN release independent of enzymatic hydrolysis [157]. This reaction was hypothesised to be used by roughly half of the polydesmid millipedes. The species *Polydesmus complanatus*, *Brachydesmus avalae* and *Brachydesmus dadayi* all contained five or six of the following compounds: benzaldehyde, benzoic acid, benzonitrile, benzoyl ethyl ketone, benzyl alcohol, benzyl methyl ketone, mandelonitrile and mandelonitrile benzoate in whole body extracts [128]. The cave-dwelling millipede *Brachydesmus troglobius* contain a mixture of benzaldehyde, benzoic acid, benzonitrile, benzyl alcohol, HCN and mandelonitrile benzoate in its secretion [127]. *Jonospeltus splendidus* contains mandelonitrile in its defensive secretion, and an unidentified CNglc together with β-glucosidase activity was also reported from this species [130]. Benzaldehyde, benzoyl cyanide and mandelonitrile were found in the glandular exudates of *A. corrugate*, *Apheloria trimaculata* and *Pseudopolydesmus serratus* [131]. *Gomphodermus pavanii* contains mandelonitrile, *Polydesmus vicinus* stores *p*-isopropyl mandelonitrile glucoside, *Polydesmus collaris* stores mandelonitrile benzoate and *Pachydesmus crassicutis* contains a mandelonitrile glucoside [10,121]. HCN was measured from the glandular secretions of *Cherokia georgiana ducilla*, *Cherokia georgiana georgiana*, *Cherokia georgiana latassa*, *Euryurus maculates*, *Motyxia tularea*, *Pseudopolydesmus erasus* and *Sigmoria nantahalae* [118]. Defence secretions from a further 17 polydesmid millipedes were analysed with GC-MS [118] and up to 35% of the secretion of the various millipedes was comprised of benzoyl cyanide and mandelonitrile benzoate [118,131]. For a full overview of cyanogenic compounds in millipedes, see Table 1.

Cyanogenesis is hypothesised to have evolved once, early in the evolution of the order of polydesmid millipedes, as a response to vertebrate predation [11], probably in parallel with the tolerant cytochrome oxidase and/or detoxification enzymes. Since the components of the defensive secretions from most polydesmid millipedes are similar and prunasin has been shown in one species, some or all of the components may have a prunasin-based system, where prunasin is present in the body and hydrolysed to mandelonitrile, which is then stored at acidic pH in the defensive glands. It has been hypothesised that centipedes, millipedes and carabid beetles (Coleoptera) have each independently evolved the common cyanogen mandelonitrile [141].

### 6.3. Arachnida

**Figure 10:**
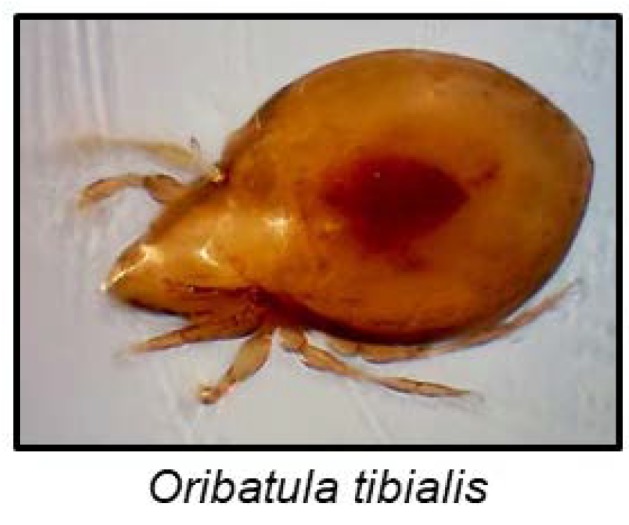
*O. tibialis* photo by Matthew Shepherd (http://www.soilbiodiversityuk.myspecies.info/taxonomy/term/16371/media).

Although arachnids are famous for their noxious chemical defences, cyanogenesis has only been reported in a single species to date [137]. The mite *Oribatula tibialis* (Figure 10) stores mandelonitrile hexanoate, a cyanogenic aromatic ester, in exocrine oil glands. After disturbance, a defensive secretion containing benzoyl cyanide, benzoic acid and hexanoic acid is emitted. In a hydrophobic environment, mandelonitrile hexanoate remained intact, but contact with moisture resulted in its hydrolyses to hexanoic acid, benzaldehyde and HCN [137]. Mandelonitrile hexanoate is probably produced from phenylalanine, and the hydrolysis to release HCN could be non-enzymatic [137].

Many mite species are able to handle CNglcs, since they are agricultural pests on cyanogenic plants, such as some species of the genera *Mononychellus* [158] and *Tetranychus* [81] feeding on plants containing aliphatic CNglcs (cassava and lima beans, respectively). These mites are able to feed on cyanogenic plants because they contain an effective β-cyanoalanine synthase for detoxification of HCN as observed in members of the order Lepidoptera (discussed in Section 4) [82].

### 6.4. Hemiptera

**Figure 11:**
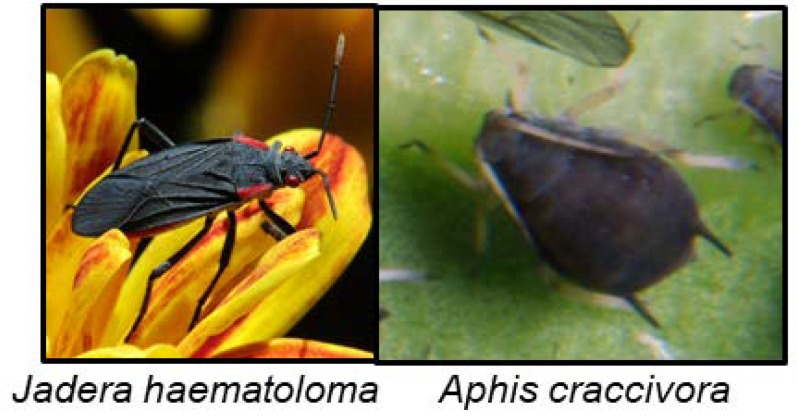
*J. haematoloma* photo by Louis J. Bradley (https://commons.wikimedia.org/wiki/File:Red_Shouldered_Bug,_Ant,_Mum.jpg) and *A. craccivora* photo by David Perez (https://commons.wikimedia.org/wiki/File:Aphis_craccivora_01_by-dpc.jpg).

CNglcs have not been found in the order Hemiptera, although other cyanogenic compounds have, and these will be briefly summarised here. The compounds are exclusively sequestered from food plants in this order, and not biosynthesised. The scentless plant bugs *Jadera haematoloma* (Figure 11) and *Jadera sanguinolenta* are able to sequester cyanolipids from the seeds of their food plants within the family Sapindaceae. When reared on food plants containing cyanolipids, they emit HCN when subsequently being crushed. When reared on acyanogenic food plants no HCN emission takes place [139]. These insects do not harbour an enzyme able to hydrolyse the cyanolipids, since HCN emission was only observed after external addition of enzymes [139]. *Leptocoris isolata* contains the cyanogenic compound cardiospermin in its larval haemolymph, which most likely is synthesised from cyanolipids sequestered from its food plant [80,140]. Cardiospermin deterred ants [140]. Adults did not contain it, perhaps again showing that expensive defence compounds are reserved for the more vulnerable life stages. The aphid *Aphis craccivora* (Figure 11) sequesters cyanamide from its food plant *Robinia pseudoacacia* (Fabaceae), which renders it toxic to its predator *Harmonia axyridis* [138]. *A. craccivora* was not toxic to *H. axyridis* if it had fed on an acyanogenic food plant. Bird cherry oat aphid (*Rhopalosiphum padi*) which feed on the cyanogenic *Prunus padus*, contained both rhodanese and β-cyanoalanine synthase activities, for detoxification of HCN [159], but it is not known if the aphid sequester and retain CNglcs in its body.

### 6.5. Coleoptera

**Figure 12:**
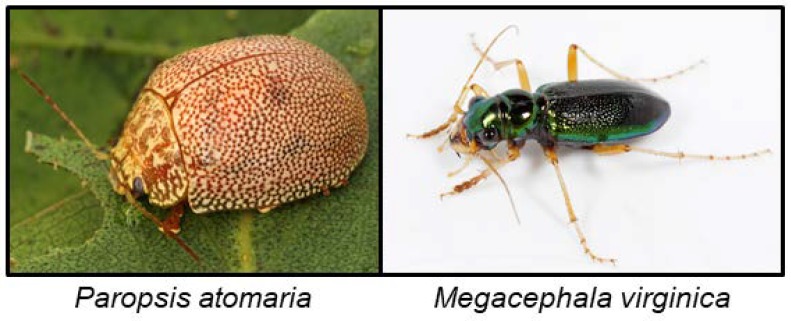
*P. atomaria* photo by Martin Lagerwey (https://commons.wikimedia.org/wiki/File:Paropsis_atomaria_Warby_Ranges2.JPG) and *M. virginica* photo by Patrick Coin (http://hasbrouck.asu.edu/neotrop/entomology/imagelib/imgdetails.php?imgid=8382).

Leaf beetles of the tribe Paropsini are widespread in Australia where they feed on plants from the genus *Eucalyptus*, of which ~4% are cyanogenic [160]. When disturbed, the beetles emit defensive secretions from vesicles on their hind body, and ants exposed to the defence secretion of *Paropsis atomaria* (Figure 12) were killed within 2 min. All life-stages of *P. atomaria* release HCN when crushed. The HCN release from adults originates from dissociation of mandelonitrile which is stable at pH 3.4 in their defence secretion [141]. All other stages of the insect are less cyanogenic than the adult and have been shown to contain the CNglc prunasin as an additional cyanogen. Since prunasin is a stable compound, it can be stored outside of the vesicles with defensive secretions, and it has been reported to be especially prevalent in life stages without defensive secretions [141]. A β-glucosidase would be needed to hydrolyse prunasin, and such activity was indeed found in larvae of *P. atomaria*. Because the food plants (*Eucalyptus blakelyi*, *Eucalyptus fastigata* and *Eucalyptus polyanthemos*) used in this study were acyanogenic, these insects are likely to biosynthesise the compounds de novo [161], and they are able to completely recharge depleted defensive vesicles within 24 h. *Megacephala virginica* (Figure 12) contains benzaldehyde, HCN and mandelonitrile [80], the first two probably being hydrolysis products of the latter. Another beetle (*Sitophilus granarius*) was shown to incorporate HCN into amino acids probably via the conversion of β-cyanoalanine [142].

### 6.6. Lepidoptera

Numerous aposematic Lepidoptera are distinctly associated with poisonous plants and may sequester toxic defence compounds from the plants sometimes in addition to manufacturing their own [3,162,163]. The ability to both biosynthesise de novo and sequester the same CNglcs appear to be restricted to Lepidopteran species. The aliphatic CNglcs linamarin and lotaustralin are especially widespread defence compounds in cyanogenic Lepidoptera, often biosynthesised in a single or more life stages. The few Lepidoptera containing aromatic CNglcs most likely sequester them from their food plants. For example, the thyridid caterpillar *Calindoea trifascialis* emits a defensive secretion, which contains benzaldehyde, benzoic acid and mandelonitrile among its constituents and serves as defence against ants [145]. Larvae of *Malacosoma americanum* (Lasiocampidae) regurgitate a droplet of enteric fluid containing benzaldehyde and HCN which successfully deter predatory ants [144]. *M. americanum* feed on cyanogenic *Prunus serotine* leaves, and preferentially feed on the young leaves as they contain the highest amount of the CNglc prunasin. Thus, *M. americanum* most likely sequester the defence compounds from the food plant to use in its defence, but the exact mechanism of this sequestration is unknown [144]. The pseudo-CNglc cycasin is present in Cycad plants and is sequestered by *Seirarctia echo* (Arctiidae) [10]. Cycasin is a glucoside which is hydrolysed in the insect gut and the aglycone is able to diffuse into tissues to be re-converted into cycasin, which remains dissolved in the insect body fluids. Despite being sensitive to HCN, larvae of the ugly nest caterpillar (*Archips cerasivoranus*, Tortricidae) and the fall webworm (*Hyphantria cunea*, Erebidae) both feed on cyanogenic cherry leaves. CNglcs could only be measured in their gut and frass and not in other body parts, indicating that they manage to excrete most of the ingested CNglcs intact, probably due to very alkaline gut pHs [164,165].

The CNglc detoxification product, β-cyanoalanine, produced by β-cyanoalanine synthase was identified in the following Lepidopteran families: Arctiidae, Cymatophoridae, Geometridae, Hesperidae, Heterogynidae, Limacodidae, Lycaenidae, Lymantriidae, Megalopygidae, Noctuidae, Notodontidae, Nymphalidae, Papilionidae, Pieridae, Yponomeutidae and Zygaenidae [78]. The amount of β-cyanoalanine was found to vary based on taxonomic, geographical and seasonal variation, which is expected since the CNglc content typically varies with the same parameters. Some species from the abovementioned groups do not contain CNglcs, but instead ingested glucosinolates, which can be detoxified by β-cyanoalanine synthases as well, at least in some pierid butterflies [83] (see Section 6.6.2). However, these butterflies evolved from ancestors feeding on plants containing CNglcs [166], which is probably why they were able to develop this glucosinolate detoxification mechanism in the first place. Other Lepidoptera containing β-cyanoalanine do not contain CNglcs (or glucosinolates), and their defensive mechanisms are based on other compounds. β-cyanoalanine is probably only produced in these species when they are detoxifying ingested CNglcs.

#### 6.6.1. Zygaenoidea

**Figure 13:**
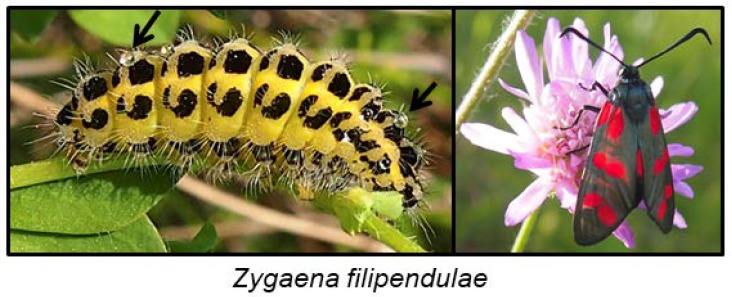
*Z. filipendulae* larva with defence droplets (arrows) and adult *Z. filipendulae* (photos by Mika Zagrobelny).

Many species from the family Zygaenidae (burnet moths) are classic examples of aposematic Lepidoptera, combining striking red and black wing patterns with potent chemical defences, based on the CNglcs linamarin and lotaustralin [167]. Linamarin and lotaustralin are present in all instars of the majority of examined species of Zygaenidae [96], and these species are extremely resistant to HCN. They are able to biosynthesise the CNglcs de novo [4,29] as well as to sequester them from larval food plants [47,168]. Larvae of the more primitive genera of Zygaeninae feed on Celastraceae, while the principal food plants of species from the genus *Zygaena* belong to the Fabaceae, Apiaceae, Asteraceae and Lamiaceae, some of which are cyanogenic [169]. Zygaenids are generally oligophagous, feeding only on one or a few plant species. *Z. filipendulae* larvae are always surrounded by a “cloud” of HCN, and it is greatly enhanced if the larvae are attacked by predators (shrews, hedgehogs, starlings, frogs and carabid beetles) [170,171,172]. Furthermore, larvae of Zygaenine species release highly viscous, colourless fluid droplets from cuticular cavities on their dorsal side (Figure 13). Contraction of irritated segments results in the appearance of droplets on the cuticular surface of the larvae [173], and the droplets may be reabsorbed a few seconds later. The droplets glue mandibles and legs of potential predators together and immobilise them. If the droplets are not reabsorbed by the larvae, they harden and form sharp crystalline-like precipitates [174]. The droplets contain the highest concentration of linamarin and lotaustralin of any tissue in the *Z. filipendulae* larva. They also contain glycine-rich peptides, proteins, glucose, and β-cyanoalanine [174]. Despite the presence of CNglcs, no specific β-glucosidase is present in the droplets, but CNglcs could deter predators by themselves since they are bad-tasting. Consequently, no HCN is released from droplets, unless they are mixed with specific β-glucosidases from the *Z. filipendulae* haemolymph or general β-glucosidases from the gut of the predator [174]. There are no gland cells or cuticular ducts leading through the cuticle into the storage cavities in *Zygaena* larva, and no special morphological adaptation for secretion has been developed in the epidermis [173]. The droplets are simply released as a result of contraction of the irritated segments in the larvae. This is in contrast to the specialised cyanogenic glands from most chilopods and diplopods [10]. *Z. filipendulae* larvae are able to retrieve CNglcs from the integument before molting, and incorporate them into the next instar, since exuviae contain only diminutive amounts of CNglcs. The detailed processes behind this phenomenon are still unknown [47,175]. The *Zygaena* sp. adult does not contain cuticular cavities but retain CNglcs from their larval stage in other tissues.

**Figure 14:**
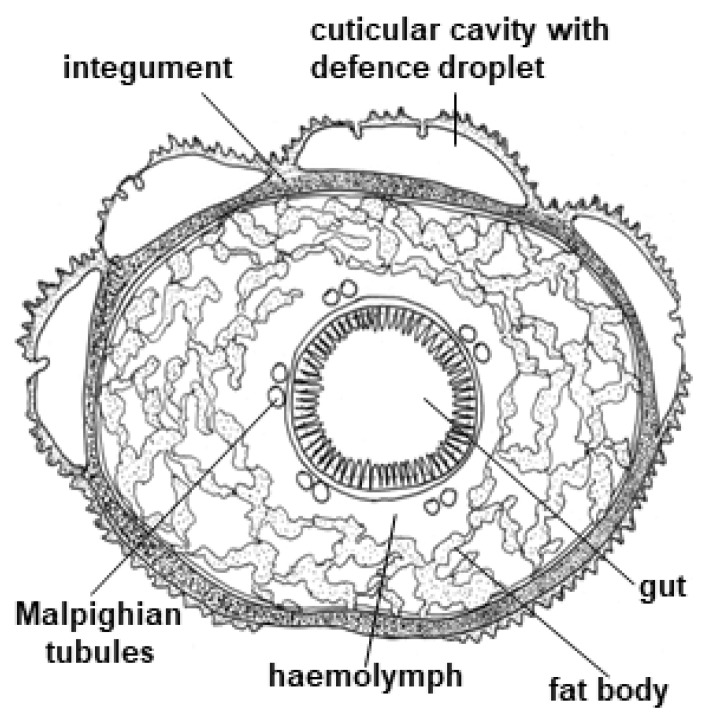
Cross section of *Z. filipendulae* larva adapted from [42].

CNglcs are produced mainly in the integument and fat body of the *Z. filipendulae* larva (Figure 14) [42]. The transcript levels of the genes and encoded proteins catalysing the biosynthetic pathway (CYP405A2, CYP332A3 and UGT33A1) are tightly regulated, being partly dependent on the content of CNglcs in the food plant [42]. Consequently, the expression of the pathway is significantly lowered when the larvae are sequestering CNglcs, but never completely turned off in feeding larvae, possibly to accommodate the polymorphic state with regards to cyanogenesis in the food plant *L. corniculatus* and/or to augment the ratio of ingested linamarin:lotaustralin. Transcription of the biosynthetic genes is turned off during pupation, resulting in a slow decline in CNglc content. Transcription is reactivated at the end of pupation in females, but not males. Eggs and embryos do not biosynthesise CNglcs, but CNglcs are transferred to them from the female before they are laid. Biosynthesis of CNglcs in female adults takes place in the integument and CNglcs are then transported to other organs, similarly to the larvae [176].

*Zygaena* larvae have β-cyanoalanine synthase activity throughout their bodies, and β-cyanoalanine accumulates in the haemolymph and integument [78]. Rhodanese activity was not found in any of the *Zygaena* species tested. All tissues in both larvae and adults of *Z. filipendulae* contain CNglcs, although ~75% of larval CNglcs reside in the integument including the cuticular cavities, and a further ~6% in emitted defence droplets. Approximately 15% resides in the haemolymph [47,177]. In adults, most CNglcs are present in abdomen, head and thorax (integument), and, in the case of females, especially in the eggs [176]. During the *Z. filipendulae* life cycle, the content and ratio of CNglcs are firmly regulated, with a 1:1 ratio of linamarin:lotaustralin in L4–L7 larval stages and at least a 2:1 ratio in subsequent stages [13]. At the different life stages, the emission of HCN and ketones resulting from the hydrolysis of CNglcs closely mirror this ratio [178]. These overall ratios cover some remarkable differences between tissues, e.g., a 1:2 ratio in defence droplets, and a 2:1 ratio in haemolymph [172]. This content and ratio is maintained relatively independent of the CNglc-composition of the food plant [47], because larvae are able to compensate by de novo biosynthesis of the deficient components. Nonetheless, larval sequestration of CNglcs from the food plant is the primary player in the overall acquisition of CNglcs in *Z. filipendulae*. Consequently, *Z. filipendulae* larvae prefer to feed on highly cyanogenic *L. corniculatus* over less cyanogenic or acyanogenic plants, probably to optimise the sequestered amount of CNglcs. When *Zygaena* larvae are forced to biosynthesise their entire content of CNglcs de novo by feeding on acyanogenic plants, their development is retarded and they show higher mortality [47]. The 1:1 ratio of linamarin:lotaustralin in the larval integument could reflect that the HCN “cloud” surrounding *Z. filipendulae* larvae is derived mainly from lotaustralin hydrolysis, since the specificity of the first enzyme in the biosynthetic pathway favours lotaustralin production [29], and some *L. corniculatus* populations contain more lotaustralin than linamarin. Linamarin may serve as a better deterrent of predators than lotaustralin since a higher amount of linamarin is present in the most vulnerable stages (adults, eggs and newly hatched larvae). This higher amount could be accomplished by a higher turn-over rate for lotaustralin in comparison to linamarin [48] during pupation, which would indeed match the specificity of the *Z. filipendulae* β-glucosidase [70].

When linamarin and lotaustralin are sequestered by *Z. filipendulae* larvae, they are taken up intact and translocated freely with the linamarin and lotaustralin originating from biosynthesis in most tissues of the insect [177]. No CNglcs are recovered in the frass [47]. *Z. filipendulae* larvae have evolved several adaptations to facilitate this sequestration, namely fast feeding, the use of a minimal disruptive feeding mode based on leaf-snipping, and a high pH in the gut [168]. Furthermore, *Z. filipendulae* saliva contains no β-glucosidases able to hydrolyse CNglcs. These adaptations are all aimed at keeping the hydrolytic enzymes (β-glucosidases and α-hydroxynitrile lyases) originating in the food plant separated from their respective substrates and thus functionally inactive during feeding and digestion. Some or all of these adaptations may be envisioned to function in other Lepidopterans as well.

Sarmentosin is a nitrile-γ-glucoside related to CNglcs, whose epoxide spontaneously releases HCN [179]. It has been hypothesised to be biosynthesised by a pathway sharing the first enzymatic step with the biosynthesis of linamarin and lotaustralin [93]. Several moth species belonging to the families Geometridae and Yponomeutidae as well as one Zygaenid (*Pryeria sinica*) contain sarmentosin. Sarmentosin was hypothesised to be sequestered from food plants in most instances but could be biosynthesised at least in Geometrid moths [3,143].

Since the Lepidopteran radiation happened much earlier and thus well separated in time from the radiation of their current food plants, the angiosperms [180], most occurrences of biosynthesis of defence compounds likely evolved before sequestration of plant products. Accordingly, the ability of Zygaenidae species to biosynthesise CNglcs [29] is probably a fundamental characteristic of Zygaenidae, perhaps evolved to deter predators, store nitrogen, or for male-female communication (see Section 9). At the same time-point or even earlier, the ability to detoxify HCN [78] would have evolved to enable the insect to evade self-intoxication. This ability would have enabled ancestors of Zygaenidae to initiate feeding on cyanogenic plants, thereby colonising a new niche of food plants mostly free of competition. The ability to sequester CNglcs from the food plant subsequently enabled *Zygaena* species to optimise their supply of CNglcs and may also be an essential and common old trait. Similarly, biosynthesis of chemicals for defence is considered a primitive state and sequestration a subsequently acquired character in Chrysomelid beetles [181].

#### 6.6.2. Papilionoidea

The aposematic butterfly *Eumaeus atala* (Lycaenidae) contain the pseudo-CNglc cycasin in all life-stages which it probably sequesters from its *Cycad* food plant *Zamia floridana* [146]. The butterfly subfamilies Nymphalinae, Polyommatinae and Heliconiinae have been reported to contain linamarin and lotaustralin similar to Zygaenoidea moths [12,48,147,148] and are also able to withstand the toxicity of these compounds [182]. However, Nahrstedt [12] did not specify in which Nymphalinae and Polyommatinae species linamarin and lotaustralin were found, and no further information on CNglcs have been reported from these subfamilies. Indeed, studies of CNglcs in butterflies have mainly been focused on the subfamily Heliconiinae.

**Figure 15:**
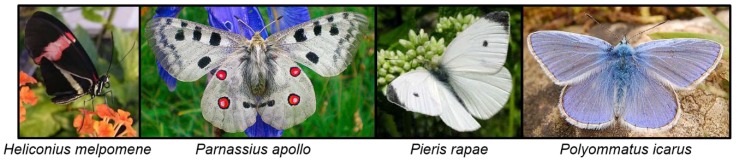
Various butterfly species. *H. melpomene* photo by Érika Cristina Pinheiro de Castro, *P. apollo* photo by Hinox (https://commons.wikimedia.org/wiki/File:Parnassius_apollo_Pirineus.JPG), *P. rapae* photo by David Hanson (http://www.neotropicalbutterflies.com/Site%20Revision/Pages/PieridPages/Pieris_rapae.html), *P. icarus* photo by Luc Viatour (https://commons.wikimedia.org/wiki/File:Butterfly_Luc_Viatour.JPG).

Most larvae of species from the Heliconiinae feed on plants containing CNglcs mainly of the cyclopentenoid type. This type of CNglc is restricted to Passifloraceae, Turneraceae and four other closely related plant families [183], and many Heliconiinae can sequester them from the plants. The subfamily Heliconiinae is divided into four tribes: Heliconiini, Acraeini, Argynniini, Vagrantini, and the genus Cethosia. Linamarin and lotaustralin were found in them all [48], and since the majority of their food plants lack these compounds, the ability to biosynthesise aliphatic CNglcs de novo would appear to be a common trait of this subfamily. Furthermore, assays with radiolabelled valine and isoleucine experimentally demonstrated that the advanced genus *Heliconius*, and other genera within the tribe Heliconiini, can biosynthesise linamarin and lotaustralin [49]. CNglcs are present in all developmental stages of Heliconiini and are thought to be responsible for their unpalatability [48] similar to Zygaenoidea. Contrary to *Zygaena* moths, *Heliconius* butterflies (Figure 15) biosynthesise CNglcs intensively as adults, as well as to a reduced degree as larvae. It has been suggested that this capacity is associated with their pollen-feeding behaviour, which provide them with extra nutrients for reproduction and longevity, freeing more resources for defence [184]. *Heliconius* have a very long adult life compared to other lepidopterans, and even to closely related butterflies (up to six months), which could justify that vigorous toxin production has shifted from the larval to the adult stage. *Argyreus hyperbius* from the tribe Argynniini was also recently shown to be able to biosynthesise linamarin and lotaustralin de novo [185]. Cyanogenesis has been poorly studied in the genus *Cethosia* and the tribe Vagrantini, but species from both are known to release HCN [49].

Heliconiini larvae feed only on *Passiflora* plants which is the largest genus of the Passifloraceae family with more than 550 species and 23 different CNglcs reported from them [186,187]. The major herbivores on *Passiflora* plants are larvae from the Helconiini tribe [149], and they have been shown to sequester the cyclopentenoid CNglc epivolkenin from the plants [188]. By feeding *H. melpomene* larvae artificial diets containing different types of CNglcs, it was shown that they can sequester not only other types of cyclopentenoid CNglcs, but also aromatic and aliphatic CNglcs (de Castro, unpublished). The genus *Euptoieta* from the Argyniini tribe use Turneraceae and Passifloraceae plants as their larval food plant. When larvae of *Euptoieta hegesia* are reared on cyanogenic plants, the adults become more unpalatable to *Anolis* lizards [189], suggesting that they sequester CNglcs from their food plant. African genera of the tribe Acraeini feed on plants containing cyclopentenoid CNglcs as larvae, most of them preferably on Passifloraceae [148]. Consequently, *Acraea horta* can sequester the cyclopentenoid CNglc gynocardin from its larval food plants when the compound is present [190], and it is likely that many other species of this tribe have this ability as well. South American species of Acraeini feed on Asteraceae plants, and it has been hypothesised that this food plant shift happened to avoid competition with heliconiine butterflies for Passifloraceae plants [191]. Many acraeine butterflies are able to biosynthesise linamarin and lotaustralin, but it is still unknown if this process is intensified in the Asteraceae-feeding genera, which cannot sequester CNglcs from their food plant.

Heliconiine butterflies have evolved efficient mechanisms to profit from the cyanogenic defences of their *Passiflora* food plants. Female adults of *H. erato*, for example, use HCN emissions from their food plants to guide them to prime egg laying locations, as they have a preference for oviposition on *Passiflora biflora* leaves with high HCN emissions [192]. When feeding on *Passiflora capsularis*, *H. erato* larvae cause only minor HCN emission, whereas the generalist lepidopteran larvae *Spodoptera litoralis* provoke a 5–8 fold higher HCN release than freshly crushed leaves of the plant [193]. Accordingly, *H. melpomene* has been shown to perform leaf-snipping while feeding, as well as having an alkaline gut pH, two traits aimed at keeping the hydrolytic enzymes inactive and ingested CNglcs intact (de Castro, unpublished). These adaptations are similar to those seen in *Z. filipendulae.* By avoiding CNglc hydrolysis, *Heliconius* larvae optimise the sequestration of CNglcs from their food plant and reduce energy costs associated with HCN detoxification.

Several species of *Parnassius* butterflies (Papilionidae) contain sarmentosin combined with small amounts of linamarin and lotaustralin. Sarmentosin was hypothesised to be sequestered from *Sedum* food plants [93,151], although some *Parnassius* species were hypothesised to biosynthesise the compound de novo (and linamarin and lotaustralin) due to very high bodily concentrations [93]. Consequently, *Parnassius* butterflies could share an orthologous pathway for the production of linamarin and lotaustralin with other butterflies from Papilionoidea (Heliconiinae) and moths from Zygaenoidea.

Several species of Pierid butterflies (Papilionoidea) that feed on glucosinolate-containing plants, have been shown to contain three β-cyanoalanine synthase enzymes with different kinetic properties toward HCN detoxification [83]. These butterflies contain an enzyme in their gut that redirects the breakdown of glucosinolates into different types of nitriles instead of isothiocyanates [194]. Some of the nitriles are further metabolised, resulting in the release of HCN, which is detoxified by β-cyanoalanine synthase and rhodanese. β-Cyanoalanine synthase activity could also be found in several other butterfly species although here rhodanese activity was scarce [83]. Pierid butterflies fed on CNglc-containing Fabales plants before they colonised plants containing glucosinolates, so they were used to detoxifying CNglcs and could therefore easily detoxify glucosinolates after evolving the ability to redirect glucosinolate breakdown [166]. The butterfly *H. melpomene* and the moth *Amyelois transitella* also each contain three copies of the β-cyanoalanine synthase enzyme, which could be an adaptation to feeding on cyanogenic plants.

Evolutionary studies within Lepidoptera have provided evidence that the superfamilies Papilionoidea (containing *Heliconius*) and Zygaenoidea (containing *Zygaena*) belong to the same clade [195]. The capacity to produce linamarin and lotaustralin may therefore have emerged in a common ancestor of these two superfamilies [37,93]. The cyanogenic compounds could initially have evolved as unique defence compounds and sequestration could have emerged to reduce the energy costs of defence when ancestors of *Zygaena* and *Heliconius* later started feeding on plants containing CNglcs. Further adaptations to lower energy costs for defence would, in an evolutionary perspective, render the insects highly dependent upon food plants containing CNglcs. This is especially evident in derived groups of *Heliconius* (specifically monophagous sara-sapho species) where sequestration of the cyclopentenoid CNglc epivolkenin is replacing biosynthesis of aliphatic CNglcs [196]. Following the evolution of the ability to biosynthesise as well as to sequester CNglcs, it is probable that arthropods would exploit CNglcs in as many ways as possible (discussed in Section 9).

## 7. Parasitoids of Cyanogenic Insects

Parasitic insects are generally thought to be one of the major factors controlling populations of phytophagous insects in nature. Attraction of parasitoids is thus part of the arms race between plants and insects [197]. Insects that are parasitic only during their immature life stages are called parasitoids and spend their entire larval life inside the host, nearly always destroying it in the end [198]. Consequently, besides facing immune responses by the host, parasitoids may also be affected by whatever chemical defence (sequestered or biosynthesised) the host may have acquired. Large scale surveys of parasitoid incidences have shown that chemically defended insects often have high levels of parasitism in the field, the parasites probably gaining fitness benefits because they are protected from their own natural enemies while inside the host [199]. Insect parasitoids are attracted to chemical stimuli associated with their hosts, and chemicals appear to play a major role at almost every level of the host selection process.

Some parasitoids have overcome the CNglc based defence in Zygaenidae and Papilionidae and may cause severe damage to populations in years when they are common [200,201]. Species of Braconidae, Ichneumonidae, Chalcididae, Torymidae, Pteromalidae and Scelionidae (Hymenoptera) are associated with Zygaenidae as primary or secondary parasitoids and are parasitic on larvae and pupae, except for Scelionidae which are parasitic on eggs [169]. Species of *Cotesia*, *Aleiodes*, *Meteorus*, *Charops* and *Alcima* oviposit into the larval stage of *Zygaena*. The larvae of these parasitoids develop inside the *Zygaena* larvae and eventually kill it. As many as 51 species of hymenopteran parasitoids (from seven families) where found to parasitise Zygaenidae in southern Poland, and seven species of hymenopteran parasitoids belonging to five families as well as the parasitoid fly *Phryxe nemea* were found in *Z. filipendulae* in Serbia [202]. All parasitoids presumably possess an active and fast-operating detoxification system to enable them to use cyanogenic insects as hosts. *Cotesia zygaenarum*, which is a parasitoid on *Zygaena* species, was shown to contain rhodanese for detoxification of CNglcs [107], while *C. tetricus*, which is a parasite of several acyanogenic Lepidoptera, contains no rhodanese [107]. This implies that *C. zygaenarum* is especially adapted to handle HCN released from its host while *C. tetricus* is not. *Zenillia* sp. (Tachinidae, Diptera) obtained from a Zygaenid host also contains rhodanese, although this parasite is not host-specific towards Zygaenids [80]. β-cyanoalanine synthase enzymes have not been found in genomes and transcriptomes from Diptera and Hymenoptera to date.

**Figure 16:**
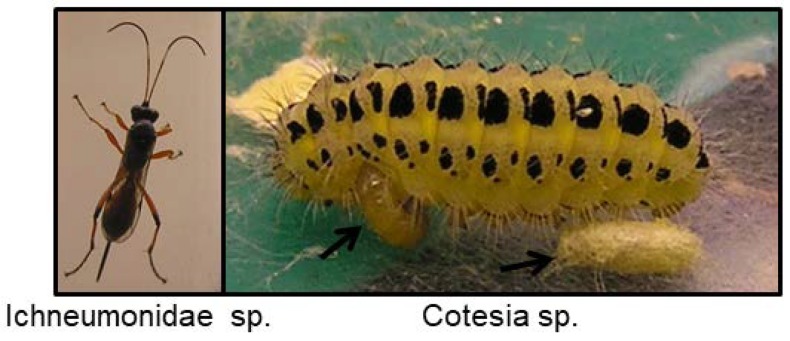
Ichneumonidae sp. emerged from a *Z. filipendulae* pupa and *Cotesia* sp. (arrows) infesting a *Z. filipendulae* larva (photos by Mika Zagrobelny).

*C. zygaenarum* cocoons have previously been shown to contain linamarin at a low level but no lotaustralin [80]. *Cotesia* larvae, which had parasitised a *Z. filipendulae* contained a 1:1 ratio of linamarin:lotaustralin at a total average level of 0.76 µg/mg f.w. (Zagrobelny, unpublished) (Figure 16). In contrast, an Ichneumonidae, which had parasitised a *Z. filipendulae* pupa, turned out to be completely acyanogenic (Zagrobelny, unpublished) (Figure 16). Parasitoids from the arctiid moth *Utetheisa ornatrix* were tested for pyrrolizidine alkaloids (PAs), which are the defence compounds of their host. PAs were absent or very low in all species of parasitoids except for the Ichneumonidae which contained one-tenth PAs (*w/w*) of that of its host [203]. This level corresponds well to the level of CNglcs found in *Cotesia* in *Z. filipendulae*, but it is probably too low to benefit the parasitoids in their own defence against enemies after they leave the host. *Cotesia congregata* which parasitises *Ceratomia catalpae* (Lepidoptera, Sphingidae) containing the iridoid glycoside catalpol was not negatively affected by the presence of the compound in their host, despite containing small amounts of the compound in their bodies in this study [204]. Parasitoids probably gain an advantage by spending their egg and larval stages within a chemically protected host even if they do not sequester enough of the toxic compound to gain protection as adults.

Colonies of *Zygaena* are extremely vulnerable to parasitoids which are not affected by their cyanogenic defence, due to very low adult dispersion. Of the few species of *Zygaena* that were studied with respect to dispersion, most individuals did not venture more than 30 m away from their home range, with a few individuals (<10%) dispersing as far away as 1 km [205]. Consequently, parasitoids can wipe out most of a *Zygaena* population and result in the size of a colony fluctuating wildly from year to year regardless of the availability of the food plant [200,201]. The ability of *Zygaena* to pass through multiple diapauses (interruption of development and reduction of metabolic activity for a time span, in this case for more than one year) [206] may be an adaptation to prevent parasitoid-induced extinction of a *Zygaena* colony, as well as to survive adverse climatic conditions.

Wasps that are parasites on eggs of heliconiine butterflies can target laid eggs or hitch-hike on mated females, where they wait to be transferred to the fresh progeny. Woelke (2008) found an egg-parasitism rate of 16.1%, and an adult hitch-hiking parasitism rate of 13.5% after collecting eggs and adults of heliconiine species in Panama. While wasps encountered in heliconiine eggs belonged to the families Encyrtidae, Scelionidae and Trichogrammatidae, adults contained individuals of a further four families: Aphelinidae, Ceraphronidae, Eulophidae and Mymaridae. It was hypothesised that some *Passiflora* species attract parasites of heliconiine eggs to protect them against these herbivores [207]. Parasitism of *Heliconius* eggs by wasps of the Vespidae and Trichommatidae families was shown to vary from 0–50%, and it was heavier when eggs were laid on *P. oerstedii* than on other *Passiflora* species, apparently because wasps were attracted to the extra floral nectar of this plant. Different *Passiflora* species also seemed to attract wasps from different families: whereas *Encytid* wasps were found more often in eggs laid on *P. auriculata*, *P. biflora* and *P. vitifolia*, Trichogrammatid wasps prefer eggs laid on *P. menispermifolia*, and *P. foetida* [208]. The egg-parasitoid *Trichogramma brassica* are attracted to benzyl cyanide produced by their *Pieris brassicae* host [209]. Males of *P. brassicae* transfer benzyl cyanide to females during mating as an anti-aphrodisiac, rendering the females less attractive to other males. *T. brassica* intercept this chemical cue to identify females close to oviposition, to be able to infect their eggs right after oviposition [209].

Polydesmid millipedes are attacked by one clade of parasitoids from the genus *Myriophora* (Diptera) despite their cyanogenic defence components. The species attracted to cyanogenic millipedes evolved from ancestors attracted to quinones, the defensive substances of other millipede orders [210].

Only few parasitoids with cyanogenic hosts have been analysed so far, and as yet no evidence regarding how they handle the ingested toxins have been brought forward. Accordingly, this is an area which would benefit greatly from future research.

## 8. Uptake and Transport of Cyanogenic Glucosides

Insects need to cope with a wide array of plant defence compounds as well as xenobiotics, which they encounter in their food plants and in the environment [1]. Sequestration is probably one of the key capacities of insect herbivores to adapt to well-defended food plants [211] and relies on the ability to keep the sequestered toxins functional. An insects’ potential to take up, transport and sequester plant derived defence compounds for their own benefit likely originates from the general nutrient uptake system. Consequently, transporters with a relatively broad specificity towards plant glucosides might be responsible for transport from the gut to the haemolymph. If food plants supply compounds matching the substrate profile of the insect transport systems, perhaps corresponding to a precursor or an intermediate involved in de novo biosynthesis of defence compounds in the insect, the insect is likely to adopt the plant compounds into their defence system. Consequently, transporters have been hypothesised to be involved in sequestration of cardiac glucosides in Danaus butterflies and iridoid glucosides in Chrysomelinae beetles [212,213]. A broad-spectrum ATP-binding cassette transporter (*Cp*MRP) was shown to be involved in the sequestration of plant derived phenolglucosides by *Chrysomela populi* [214]. *Cp*MRP acts in the defensive glands of the larvae as a pacemaker for the shuttling of metabolites from the haemolymph into defensive secretions. In plants, transporters for glycosylated defence chemicals such as glucosinolates [215] and the CNglc dhurrin [216] have also been characterised.

**Figure 17:**
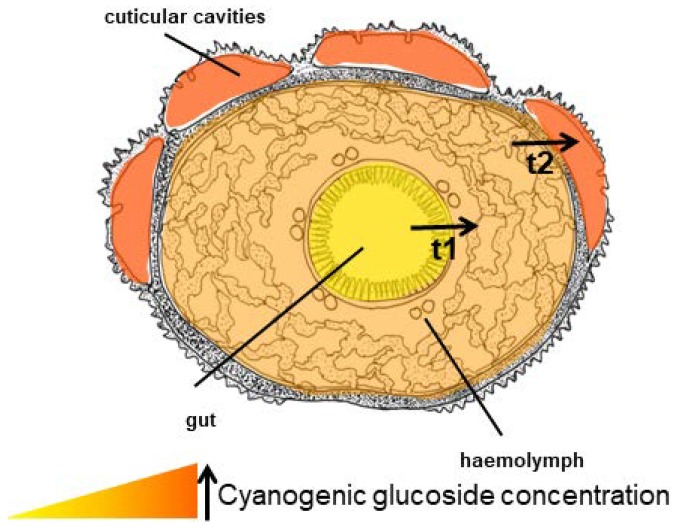
Cross-section of a *Z. filipendulae* larva showing the increasing concentration of CNglcs from the gut to the cuticular cavities, and the probable transport systems (t1 and t2).

The ability of *Z. filipendulae* to take up intact CNglcs from the food plant and rapidly distribute them to all tissues [177], and the fact that the concentration of CNglcs in the haemolymph is approximately 50 times higher than in the ingested plant material, makes it likely that *Z. filipendulae* employ one or more transport proteins for uptake. The CNglc concentration in the defensive fluid is further 5–10 times higher than the haemolymph, so transport through the skin and into the cuticular cavities also proceeds against a concentration gradient [177]. Consequently, two systems could have evolved in this insect, one for transport of CNglcs across the gut and one for transport into cuticular cavities for storage (Figure 17). Since *Z. filipendulae* can take up aromatic CNglcs [113] as well as other aliphatic hydroxynitrile glucosides [47] and *Heliconius* butterflies can sequester all three types of CNglcs (de Castro, unpublished), transporters with a relatively broad substrate specificity are probably active in the gut of these lepidopterans. However, the transporter in the gut of *Z. filipendulae* was at least specific for β-glucosides since only these were sequestered, while α-glucosides were excreted [177].

The ability of an insect to sequester plant bioactive compounds may be an important prerequisite to food plant shifts [3,217]. Within Zygaenoidea, the ability to biosynthesise CNglcs de novo is an ancient characteristic, and sequestration of CNglcs from food plants has been hypothesised to be almost as old [218]. These predispositions probably facilitated a food plant shift from Celastraceae to cyanogenic Fabaceae [219]. Similarly, de novo biosynthesis of CNglcs in *Heliconius* butterflies (Papilionoidea) has most likely facilitated the colonisation of and subsequently sequestration from cyanogenic food plants [80,196]. Likewise, some leaf beetle species have evolved to sequester pyrrolizidine alkaloids from their food plants probably due to a prior ability to synthesise and store triterpene saponins and cardenolides [220,221]. In species of sawfly (Hymenoptera), food plant shifts from iridoid glucoside-containing Lamiales to glucosinolate containing Brassicales was probably facilitated by a broadening of the ability to sequester and handle glucosides in the Brassicales feeders, which are still able to sequester iridoid glucosides as well as glucosinolates, whereas the Lamiales feeders cannot sequester glucosinolates [222].

## 9. Roles apart from Defence for Cyanogenic Glucosides and Their Hydrolysis Products

Whenever CNglcs are found in an arthropod, they have been hypothesised to be involved in defence against predators. However, CNglcs are very stable compounds due to the attached glucose molecule, rendering them attractive storage products for carbohydrates and reduced nitrogen, a function which has been shown in plants [18,19,20,21]. Furthermore, mandelonitrile has been shown to be metabolised into the plant hormone salicylic acid in peach [22], highlighting how natural products can be intimately linked to primary metabolism. Furthermore, the volatiles emitted when CNglcs are hydrolysed could easily be envisioned to have a function in inter- and intraspecific communication in insects. It would be advantageous to sub-contract such expensive defence compounds for other purposes in the insect life-cycle. Indeed, CNglcs have been shown to be transferred from the male to the female as a nuptial gift deposited in the spermatophore in both *Zygaena* moths [172,177] and *Heliconius* butterflies [223]. Nuptial gifts are widespread in insects and comprise food items or glandular products present in the spermatophore offered as paternal investment in offspring and/or to promote mating [224,225]. Male *Z. filipendulae* transfer ~30% of their body mass, including ~30% of their CNglc content [226], to females during mating. The CNglc content is mainly used for the females own defence, with only small amounts transferred to her eggs [177]. The compounds transferred in the nuptial gift are probably not essential to female *Zygaena* since they are capable of sequestering and biosynthesising CNglcs themselves as larvae. However, they do become depleted by egg-laying, so perhaps the compounds are used to top up their own reserves of defence compounds. Female *Z. filipendulae* resist mating with males with a low content of CNglcs [172] but will accept these males after they have been injected with linamarin or lotaustralin [178], when tested in the laboratory. This indicates that females are able to assess how much CNglc each male can contribute to her. Such an assessment system has been found in *U. ornatrix*, which contain pyrrolizidine alkaloids for chemical defence. Males convert some of these compounds into a volatile pheromone which is then exposed to females on their abdominal brushes. The amount of pyrrolizidine alkaloids carried by each male is honestly reflected in the concentration of the converted compound. Consequently, females are able to assess the amount of defence compound and to select males with the potential to donate high amounts to her [227,228,229]. Adult *Zygaena* males also have abdominal brushes and these have been shown to contain higher levels of HCN and CNglcs after mating, perhaps as a residual left over from courtship, so a similar system could be in effect here [178]. Since a male in poor health would probably not be able to sequester/produce large amounts of CNglcs, the purpose of the female assessment could be to observe the health and vigour of the male and thereby his ability to father healthy offspring.

Virgin females emit somewhat higher levels of HCN, acetone and 2-butanone than mated females, indicating a role of these hydrolysis products of linamarin and lotaustralin in female calling behaviour [178]. HCN was not emitted during copulation, so clearly CNglcs are not important for defence at this life stage. In many arthropods, defence compounds serve an additional function as pheromones, probably to conserve energy [155,228], so the notion of HCN serving as a pheromone in *Zygaena* is likely. If the female use HCN, acetone and/or 2-butanone to attract males, the nuptial gift of CNglcs from the male could be important to replenish her reserves, especially if she mates more than once, which *Z. filipendulae* adults have occasionally been observed to do.

During metamorphosis from larvae to adult in *Zygaena* 55% of their CNglcs are lost and the linamarin:lotaustralin ratio shifts dramatically. Since frass, exuvie and cocoons contain few or no CNglcs, the loss cannot be explained by excretion [47]. Emission of HCN and ketones resulting from the hydrolysis of CNglcs for defensive purposes is also not quantitatively important during pupation [172,178]. The emission of HCN and other hydrolysis products of CNglcs are not continuous during pupation but happen during brief periods of the transformation phase [4,178]. Since the nitrogen-containing polymer chitin is produced in quantity during insect metamorphosis, the nitrogen could be acquired by rapid turn-over of the CNglcs stored in the Zygaena larva. This would permit remobilisation of reduced nitrogen and offer an explanation for the observed loss of CNglcs during the transition phase. If cyanoalanine hydratases convert β-cyanoalanine derived from detoxification of CNglcs into asparagine and aspartic acid, as has been shown in plants, this could be achieved [74,76]. Asparagine and aspartic acid could be further processed by transaminases or proline or glutamate dehydrogenases which are active in the fat body or haemolymph of some insects [230,231,232]. Eventually the nitrogen could be incorporated into chitin via the precursor *N*-acetyl-glucosamine formed by the activity of glutamine-fructose-6-phosphate aminotransferase [233]. The observed brief periods of CNglc hydrolysis during pupation indicate that the compounds could be used at critical points during development contrary to providing a continuous cloud of HCN for defence. β-Cyanoalanine was found in large amounts in the haemolymph and defence droplets in *Z. filipendulae*, rendering an internal turn-over of CNglcs likely [174]. Lotaustralin could be the preferred substrate for the hydrolytic enzymes and therefore more amenable to turn-over in comparison to linamarin which could explain the changes in the ratio of CNglcs during the *Zygaena* life cycle. Alternatively, linamarin could be protected from use as a nitrogen source because it is favoured as the most effective defence compound for adults and eggs.

The common blue butterfly, *Polyommatus icarus* (Lycaenidae), was also hypothesised to utilise the nitrogen (and possibly glucose) from CNglc hydrolysis during its development [234]. Larvae gain more weight and have a faster development when fed cyanogenic compared to acyanogenic *L. corniculatus* leaves [234]. Feeding on cyanogenic leaves furthermore afforded increased pupal and adult weight. Two *P. icarus* specimens caught as they were nectaring on *L. corniculatus* containing linamarin and lotaustralin, were found to contain small amounts of linamarin and lotaustralin as well (Zagrobelny, unpublished), corroborating that these butterflies can take up CNglcs from their food plants. *Heliconius sara* was reported to convert the sequestered cyclopentenoid CNglc epivolkenin into the non-cyanogenic compound sarauriculatin, by replacing the nitrile with a thiol group [188]. This would allow the released nitrogen to be used for other metabolic processes.

The natural compounds including the CNglcs present in the secretory components from millipedes, were suggested to play a role in intraspecific communication as well as in defence [127], because the hydrolysis products of the CNglcs are volatile. Furthermore, low concentrations of benzaldehyde caused millipedes to aggregate, while high concentrations made them disperse [11].

In conclusion, CNglcs have acquired multiple physiological functions especially in moths and butterflies which are manifested throughout the entire life cycle. Such functions could also be envisioned to operate in other less well studied arthropods. Evidence for the involvement of CNglcs in other processes than defence have only been found in a few arthropod species (Table 2), and in some cases only as circumstantial evidence. It is also not known if all three types of CNglc (aliphatic, aromatic and cyclopentenoid) can participate in these alternative roles. Accordingly, this is an area in need of extensive research, where important future discoveries are bound to be made.

## 10. Conclusions

(1) CNglcs are important defence compounds in many arthropods, and examination of more arthropod species may disclose that CNglcs are even more widespread than currently envisioned. A defence function is only one among several possible roles of the compounds. It is reasonable to think that, when an insect has developed a mechanism to produce, sequester and store defence compounds, it would find additional uses for the stored compound to gain improved fitness and to conserve energy. In arthropods, a function as pheromones could be envisioned for the volatile hydrolysis products of CNglcs, and intact CNglcs may be used as nuptial gifts to ensure better protection of the mate and offspring. Both roles have been demonstrated in some arthropod species. Since CNglcs are nitrogen containing compounds which upon the operation of an endogenous turn-over pathway may serve as readily mobilizable storage deposits of reduced nitrogen, CNglcs may have a role in primary metabolism as nitrogen buffers. Such a function is known from plants and has also been hypothesised in one moth and one butterfly species. Another aspect is the functional roles of CNglcs in the ongoing arms race between insects and predators and/or parasitoids. As many predators and parasitoids in time learn to overcome defence compounds, elaborate systems of biosynthesis, sequestration and detoxification of the compounds will not become obsolete if the defence compounds have acquired new roles in the life cycle of the insect. Compounds that initially served defence purposes may now acquire new functions in intraspecific communication, host–insect recognition or be recruited as storage compounds that are mobilised when needed to counteract imbalances in primary metabolism.
(2) The ability to biosynthesise and hydrolyse CNglcs de novo has evolved independently in arthropods and plants. In arthropods, this even happened multiple times and at very different time points. In this context, it is remarkable that the biochemical pathways for synthesis and hydrolysis are so similar at the global level. This indicates that there is only one efficient universal route by which CNglcs can be produced and hydrolysed. This has guided a highly selective recruitment of the demanded enzyme activities. The broad distribution of CNglcs among plants and arthropods serves to demonstrate their overall beneficial functions for the host organism. Likewise, although the intermediates in their biosynthesis are numerous and chemically highly unusual, their biosynthesis from amino acids is catalysed by just three enzymes. Similarly, hydrolysis of CNglcs or detoxification of liberated HCN demands the operation of only a single or two enzymes.
(3) In parallel with the evolution of the biosynthetic pathway of CNglcs, ways to handle the toxic compounds formed, e.g., with respect to transport, storage and detoxification, would have to evolve. In the case of herbivores, the detoxification mechanism had probably already evolved in a distant ancestor, since all herbivores encounter HCN to a low degree upon ingestion of the food plant material. In the case of Lepidoptera, a more effective system evolved with the recruitment of the bacterial β-cyanoalanine synthase. This enabled many species to commence feeding on highly cyanogenic plants. Such arthropods already equipped with the necessary mechanisms to handle CNglcs would have been perfectly prepared to start sequestering CNglcs from food plants. This minimises the energy spent for defence and provides a separate environmental niche in which the competition from other herbivores may be less pronounced.
(4) Much knowledge regarding the presence, biosynthesis and hydrolysis of CNglcs in arthropods has emerged within recent years. However, key knowledge on CNglcs is still missing regarding their distribution, their roles apart from defence and their cellular and sub-cellular storage sites. The convergent evolution of CNglc metabolism remains another open area for future studies. We hope this review will spur further interest in these topics and stimulate research into these questions.

## Figures and Tables

**Table 1:** Cyanogenesis in Arthropoda.

Class/Order	Superfamily/Family	Species	Cyanogenic Component (Type)	References
Chilopoda	Scolopendridae	*Asanada* sp.	HCN	[114]
	Geophilidae	*Geophilus vittatus*	Benzaldehyde, benzoic acid benzoyl cyanide, HCN, mandelonitrile (aromatic)	[115]
		*Pachymerium ferrugineum*	HCN	[114]
	Himantariidae	*Himantarium gabrielis*	benzaldehyde, benzoyl cyanide, benzyl cyanide, HCN, mandelonitrile, mandelonitrile benzoate (aromatic)	[116]
Diplopoda	Cryptodesmidae	*Niponia nodulosa*	Benzaldehyde, benzoic acid, benzoyl cyanide, mandelonitrile, mandelonitrile benzoate (aromatic)	[117]
	Euryuridae	*Euryurus maculates*, *Euryurus leachii*	Benzaldehyde, HCN (aromatic)	[118,119]
	Gomphodesmidae	*Astrodesmus laxus*	Benzaldehyde, HCN (aromatic)	[120]
		*Gomphodermus pavanii*	benzaldehyde, benzoic acid, HCN, mandelonitrile, mandelonitrile benzoate (aromatic)	[10,11,121]
	Paradoxosomatidae	*Chamberlinius hualienensis*	Benzaldehyde, benzoic acid, HCN, mandelonitrile, methyl benzoate (aromatic)	[11,72]
		*Neodyopus patrioticus*	Benzaldehyde, HCN, mandelonitrile (aromatic)	[122]
		*Orthomorpha coarctata*	Benzaldehyde, benzoic acid, HCN (aromatic)	[123,124]
		*Oxidus gracilis*	Benzaldehyde, benzoic acid, ethyl benzoate, HCN, mandelonitrile (aromatic)	[123,125]
		*Streptogonopus phipsoni*	Benzaldehyde (aromatic)	[126]
	Polydesmidae	*Brachydesmus troglobius*	Benzaldehyde, benzoic acid, benzonitrile, benzyl alcohol, HCN, mandelonitrile benzoate (aromatic)	[127]
		*Brachydesmus avalae*, *Brachydesmus dadayi*	Benzaldehyde, benzonitrile, benzoic acid, benzoyl ethyl ketone, benzyl alcohol, benzyl methyl ketone, mandelonitrile, mandelonitrile benzoate (aromatic)	[128]
		*Epanerchodus japonicus*	Benzaldehyde, HCN, mandelonitrile (aromatic)	[129]
		*Jonospeltus splendidus*	Mandelonitrile (aromatic)	[130]
		*Polydesmus complanatus*	Benzaldehyde, benzonitrile, benzoic acid, benzoyl ethyl ketone, benzyl alcohol, benzyl methyl ketone, mandelonitrile, mandelonitrile benzoate (aromatic)	[128]
		*Polydesmus vicinus*	*p*-isopropyl mandelonitrile glucoside (aromatic)	[10,121]
		*Polydesmus collaris*	Mandelonitrile benzoate (aromatic)	[10,121]
		*Pseudopolydesmus canadensis*	HCN	[120]
		*Pseudopolydesmus erasus*	Benzaldehyde, ethyl benzoate HCN, mandelonitrile benzoate (aromatic)	[118]
		*Pseudopolydesmus serratus*	Benzaldehyde, benzoyl cyanide, mandelonitrile, mandelonitrile benzoate (aromatic)	[131]
	Xystodesmidae	*Apheloria trimaculata*	Benzaldehyde, benzoyl cyanide, mandelonitrile (aromatic)	[131]
		*Cherokia georgiana ducilla*, *Cherokia georgiana georgiana*, *Cherokia georgiana latassa*	Benzaldehyde, benzoic acid, benzoyl cyanide, HCN, mandelonitrile benzoate (aromatic)	[118]
		*Cleptoria rileyi*	Benzaldehyde, benzoic acid, benzoyl cyanide, HCN, mandelonitrile benzoate (aromatic)	[118]
		*Harpaphe haydeniana*	Benzaldehyde, HCN, mandelonitrile (aromatic)	[10,132]
		*Motyxia tularea*, *Motyxia tiemanni*, *Motyxia sequoiae*	Benzaldehyde, benzoic acid, benzoyl cyanide, HCN, mandelonitrile benzoate (aromatic)	[118]
		*Nannaria* sp.	HCN	[120]
		*Pachydesmus crassicutis*	Benzaldehyde, HCN, mandelonitrile glucoside (aromatic)	[10,121]
		*Paimokia* sp.	Benzaldehyde, benzoyl cyanide, HCN (aromatic)	[118]
		*Parafontaria laminate armigera*	Benzaldehyde, benzoate, benzoic acid, benzoyl cyanide, mandelonitrile (aromatic)	[133]
		*Parafontaria tonominea*	Benzaldehyde, HCN, mandelonitrile (aromatic)	[134]
		*Sigmoria nantahalae*	Benzaldehyde, benzoic acid, benzoyl cyanide, HCN (aromatic)	[118]
		*Stelgipus agrestis*	Benzaldehyde, benzoyl cyanide, HCN (aromatic)	[118]
		*Rhysodesmus vicinus*	HCN, *p*-isopropyl mandelonitrile (aromatic)	[135]
		*Riukiaria pugionifera*	Benzaldehyde, benzoic acid, HCN, mandelonitrile, (aromatic)	[136]
		*Riukiaria semicircularis semicirkularis*	Benzaldehyde, benzoyl cyanide, HCN, mandelonitrile, mandelonitrile benzoate (aromatic)	[134]
Arachnida	Oripodoidea	*Oribatula tibialis*	Benzaldehyde, HCN mandelonitrile hexanoate (aromatic)	[137]
Hemiptera	Aphidoidea	*Aphis craccivora*	Cyanamide (other)	[138]
	Rhopalidae	*Jadera haematoloma*	Cyanolipids, HCN (other)	[139]
		*Jadera sanguinolenta*	Cyanolipids, HCN (other)	[139]
		*Leptocoris isolata*	Cardiospermin (other)	[80,140]
Coleoptera	Carabidae	*Megacephala virginica*	Benzaldehyde, HCN, mandelonitrile (aromatic)	[80]
	Chrysomelidae	*Paropsis atomaria*	HCN, mandelonitrile, prunasin (aromatic)	[141]
	Curculionidae	*Sitophilus granarius*	HCN	[142]
Lepidoptera	Arctiidae	*Seirarctia echo*	Cycasin (other)	[10]
	Geometridae	*Abraxas grossulariata*	Sarmentosin (other)	[143]
	Lasiocampidae	*Malacosoma americanum*	Benzaldehyde, HCN (aromatic)	[144]
	Thyrididae	*Calindoea trifascialis*	Benzaldehyde, benzoic acid, mandelonitrile (aromatic)	[145]
	Yponomeutidae	*Yponomeuta hexabolus*	Sarmentosin (other)	[3]
	Zygaenoidea	*Majority of examined species from Zygaenidae*	Linamarin, lotaustralin (aliphatic)	[96]
		*Pryeria sinica*	Sarmentosin (other)	[3]
	Papilionoidea	*Eumaeus atala*	Cycasin (other)	[146]
		*Subfamilies Nymphalinae*, *Polyommatinae*, *Heliconiinae*	Linamarin, lotaustralin (aliphatic), prunasin (aromatic), epivolkenin and various cyclopentenoid CNglcs	[12,48,147,148,149,150]
		*Parnassius apollo*, *Parnassius smintheus*	Linamarin, lotaustralin (aliphatic), sarmentosin (other)	[93]
		*Parnassius phoebus*	Sarmentosin (other)	[151]

**Table 2:** Roles of CNglcs apart from defence in Arthropoda.

Role	Cyanogenic Component (Type)	Species (Order)	References
Storage and mobilisation of reduced nitrogen (and glucose)	Linamarin and lotaustralin (aliphatic) Epivolkenin (cyclopentenoid)	*Z. filipendulae*, *P. icarus*, *H. sara* (Lepidoptera)	[4,47,188,234]
Intraspecific communication	Hydrolysis products of prunasin or mandelonitrile (aromatic)	Millipedes (Diplopoda)	[11,127]
Pheromone	Linamarin and lotaustralin (aliphatic)	*Z. filipendulae* (Lepidoptera)	[178]
Assessment of mate quality	Linamarin and lotaustralin or their hydrolysis products (aliphatic)	*Z. filipendulae* (Lepidoptera)	[172,178]
Nuptial gift	Linamarin and lotaustralin (aliphatic)	*Z. filipendulae*, 9 species of *Heliconius* (Lepidoptera)	[172,177,223]
